# Structure-activity relationships for analogs of the tuberculosis drug bedaquiline with the naphthalene unit replaced by bicyclic heterocycles

**DOI:** 10.1016/j.bmc.2018.02.026

**Published:** 2018-05-01

**Authors:** Hamish S. Sutherland, Amy S.T. Tong, Peter J. Choi, Daniel Conole, Adrian Blaser, Scott G. Franzblau, Christopher B. Cooper, Anna M. Upton, Manisha U. Lotlikar, William A. Denny, Brian D. Palmer

**Affiliations:** a Auckland Cancer Society Research Centre, School of Medical Sciences, University of Auckland, Private Bag 92019, Auckland 1142, New Zealand; b Maurice Wilkins Centre, University of Auckland, Private Bag 92019, Auckland 1142, New Zealand; c Institute for Tuberculosis Research, College of Pharmacy, University of Illinois at Chicago, 833 South Wood Street, Chicago, IL 60612, USA; d Global Alliance for TB Drug Development, 40 Wall St, New York, NY 10005, USA

**Keywords:** Bedaquiline, Bedaquiline analogs, Tuberculosis, Drug development

## Abstract

Replacing the naphthalene C-unit of the anti-tuberculosis drug bedaquiline with a range of bicyclic heterocycles of widely differing lipophilicity gave analogs with a 4.5-fold range in clogP values. The biological results for these compounds indicate on average a lower clogP limit of about 5.0 in this series for retention of potent inhibitory activity (MIC_90_s) against *M.tb* in culture. Some of the compounds also showed a significant reduction in inhibition of hERG channel potassium current compared with bedaquiline, but there was no common structural feature that distinguished these.

## Graphical abstract

**Figure:**
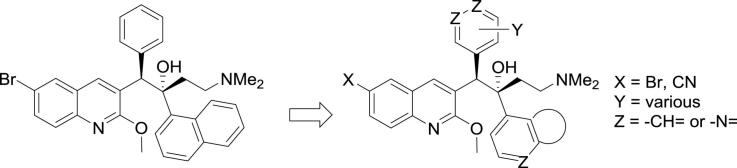

## 1 Introduction

**Fig. 1:**
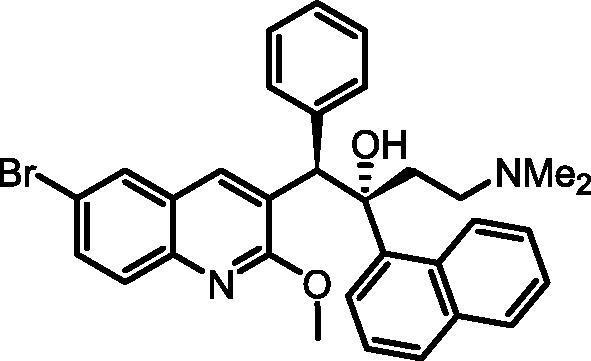
Bedaquiline (**1**).

Bedaquiline (TMC207, Sirturo®, Janssen Pharmaceuticals; Fig. 1; **1**) is an exciting new drug for the treatment of tuberculosis (TB). It exhibits a novel mechanism of action compared to other TB drugs namely inhibition of the ATP synthase^1^ of *Mycobacterium tuberculosis* (*M.tb*), the etiological agent of TB. Resistance to other TB drugs occurs primarily due to mutations in genes encoding their respective bacterial drug targets. Due to its novel drug target, bedaquiline is active against *M.tb* strains resistant to other drugs, and demonstrates efficacy against multi-drug-resistant (MDR) TB.^2^ Bedaquiline was approved by the US FDA in 2012 for specific use in pulmonary MDR-TB, and does show improved outcomes when it is used in combination with standard MDR-TB drugs.^3^ A recent study adding bedaquiline to a standard regimen for the treatment of MDR-TB showed a positive cost-benefit analysis by shortening hospital stays.^4^ Potential drawbacks of bedaquiline include inhibition of the hERG (human Ether-à-go-go-Related Gene; KCNH2) potassium channel^5^ (with the concomitant risk of cardiac toxicity), hepatic toxicity,^6^ and possibly a risk of phospholipidosis^7^ (related to its high lipophilicity [calculated clogP of 7.25]).^8^ There are also potential pharmacokinetic (PK)-based drug-drug interactions with the common TB drugs rifampicin and rifapentine, which are potent inducers of CYP3A4, the major metabolising enzyme for bedaquiline.^9^

The development of analogs of bedaquiline designed to improve on some of these properties is thus of high interest. We have previously explored the effects of more polar 6-substituents on the quinoline ring^10^ and replacement of the unsubstituted phenyl ring with various heterocycles.^11^ In this paper, we explore the effects of a variety of (mostly) more hydrophilic bicyclic C units (Fig. 1) in place of the naphthalene unit of bedaquiline. In addition to being another way to lower the overall lipophilicity of analogs, a particular interest in exploring unit C structure is highlighted by a recent paper^12^ on the 1.7 Å resolution crystal structure of bedaquiline bound to the c subunit of the ATP synthase F_o_ of the mycobacterium *M. phlei* (84% sequence identity with *M.tb*). This shows the dimethylaminoethyl unit D making an H-bond to Glu65 in the ion-binding site of the enzyme, with the rest of the molecule demonstrating multiple hydrophobic contacts with the enzyme, including the naphthalene unit C with Tyr68 and Leu72, but clashing with Tyr70. Bicyclic alternatives to the naphthalene are thus of interest from a structural point of view, and in this paper, we prepare and evaluate a series of these, with the major focus on less lipophilic examples than naphthalene.

## 2 Results and discussion

### 2.1 Chemistry

**Scheme 1:**
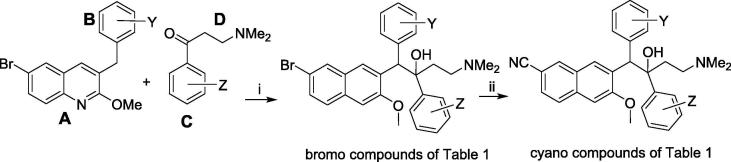
Synthesis of the compounds of Table 1. *Footnote for Scheme 1*. (i) (a) HN(iPr)_2_, *n*-BuLi, THF, −40 °C, 0.25 h; (b) A/B-unit, THF, −78 °C, 1.5 h; (c) Mannich base, THF, −78 °C, 4 h; (ii) Zn, Zn(CN)_2_, Pd_2_(dba)_3_, P(o-tol)_3,_ DMF, 50 °C.

**Scheme 2:**
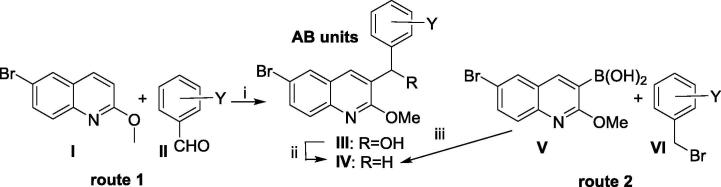
Synthesis of the required A/B units. *Footnote for Scheme 2*. Y = Me (Ref. 8): Y = H; 3-F; 2-F, 3-OMe; 2,3-diOMe (Ref. 10): 3-aza, 2-OMe; 3-aza, 4,5-diOMe; 4-aza, 2,3-diOMe (Ref 11); Y = 2,3-O(CH_2_)_2_O– (route 2, yield 52%); 4-aza, 3,5-diOEt (route 2, yield 56%); 4-aza, 3-OMe, 5-OEt (route 2, yield 68%); 4-aza, 3-OMe, 5-OiPr (route 2, yield 78%) 4-aza, 3-OMe, 5-OnPr (route 2, yield 75%), 4-aza, 3-OEt, 5-OnPr (route 2, yield 78%) (this paper). (i) LiTMP, then AcOH; (ii) Et_3_SiH, TFA, DCM, 20 °C; (iii) Cs_2_CO_3_, Pd(PPh_3_)_4_, PhMe/DMF, 110 °C (sealed tube), 5 h.

The 6-bromo compounds of Table 1 were prepared as previously described,^8^, ^10^ by LiTMP/LDA-mediated coupling of appropriate benzylquinoline A/B-units and 3-(dimethylamino)-1-phenylpropan-1-one (Mannich base) C/D-units. The cyano derivatives were prepared by Pd-mediated cyanation of the corresponding bromo analogs (Scheme 1). The resulting diarylquinolines were formed as a racemic mixture of four diastereomers, and the desired *RS*,*SR* diastereomer (depicted) was isolated by super-critical fluid HPLC at BioDuro LLC (Beijing). Twelve different A/B units were used, apart from the unsubstituted parent; syntheses of many of these been reported previously.^10^, ^11^ The remainder were prepared as outlined in Scheme 2. In previous work,^10^ we noted that the yields in the final condensation reaction (Scheme 1) seemed to correlate positively with the expected degree of electron density at the benzylic position of the AB-subunit, but this was not obvious in the present study.

**Table 1:** Bicyclic “C-unit” (Z) analogs of bedaquiline.

**Figure:**
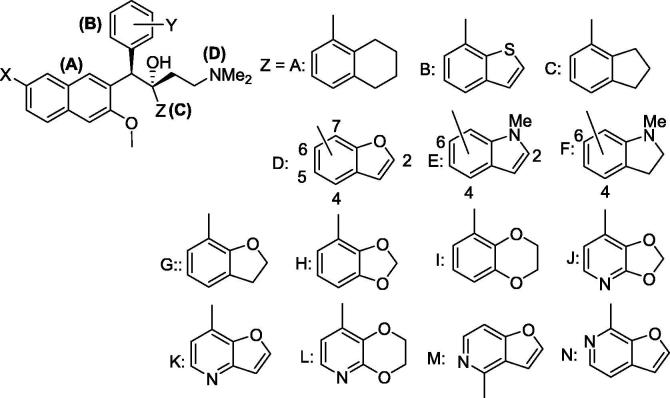

No	X	Y	Z	Yld^a^	MIC_90_^b^ (µg/mL)		clogP^c^
					MABA	LORA	
**1**					0.07	0.11	7.25
**2**	Br	3-Me	A	32	0.04	0.03	8.15
**3**	CN	3-Me	A	52#	0.09	0.06	6.79
**4**	Br	3-F	A	8	0.04	0.05	7.79
**5**	Br	H	B	38	0.02	0.04	7.11
**6**	CN	H	B	84#	0.07	0.12	5.75
**7**	Br	H	C	65	0.07	0.08	7.09
**8**	CN	H	C	83#	0.15	0.09	5.73
**9**	Br	2,3-diOMe	C	49	0.05	0.07	6.35
**10**	CN	2,3-diOMe	C	54#	0.07	0.07	4.99
**11**	Br	2-F, 3-OMe	C	46	<0.02	0.08	7.09
**12**	Br	2,3-O(CH_2_)_2_O–	C	42	0.015	0.008	7.01
**13**	Br	4-aza, 2,3-diOMe	C	77	<0.02	<0.02	6.06
**14**	Br	H	D-7	33	0.06	0.07	6.64
**15**	CN	H	D-7	72#	0.12	0.08	5.28
**16**	CN	3-F	D-7	73#	0.04	0.08	5.42
**17**	CN	3-Me	D-7	76#	0.08	0.13	5.78
**18**	Br	3-aza, 2-OMe	D-7	38	0.49	0.55	5.56
**19**	CN	3-aza, 2-OMe	D-7	48#	0.60	0.54	4.20
**20**	Br	3-aza, 2,5-diOMe	D-7	68	0.02	0.06	6.43
**21**	Br	3-aza, 4,5-diOMe	D-7	27	0.93	1.1	6.48
**22**	Br	4-aza, 2,3-diOMe	D-7	45	0.06	0.13	5.61
**23**	CN	4-aza, 2,3-diOMe	D-7	75#	0.27	0.30	4.25
**24**	Br	4-aza, 3,5-diOEt	D-7	83	0.02	0.03	7.41
**25**	CN	4-aza, 3,5-diOEt	D-7	36#	<0.02	0.03	6.06
**26**	Br	4-aza, 3-OMe, 5-OEt	D-7	65	<0.02	<0.02	6.87
**27**	CN	4-aza, 3-OMe, 5-OEt	D-7	48#	<0.02	0.07	5.53
**28**	Br	4-aza, 3-OMe, 5-O^i^Pr	D-7	65	<0.02	<0.02	7.19
**29**	CN	4-aza, 3-OMe, 5-O^i^Pr	D-7	50#	<0.02	0.08	5.84
**30**	Br	4-aza, 3-OMe, 5-O*^n^*Pr	D-7	37	0.01	0.01	7.42
**31**	CN	4-aza, 3-OEt, 5-O*^n^*Pr	D-7	49#	0.02	0.05	6.06
**32**	Br	H	D-6	50	0.04	0.13	6.64
**33**	CN	H	D-6	78#	2.3	2.2	5.28
**34**	Br	H	D-5	53	0.11	0.16	6.64
**35**	CN	H	D-5	76#	0.19	0.18	5.28
**36**	Br	4-aza, 2,3-diOMe	D-5	63	0.03	0.02	5.61
**37**	Br	4-aza, 2,3-diOMe	D-4	48	<0.004	0.007	5.61
**38**	Br	4-aza, 3-OMe,5-OEt	D-4	52	0.01	0.01	6.89
**39**	Br	4-aza, 3-OMe,5-O^i^Pr	D-4	40	0.01	0.01	7.20
**40**	CN	4-aza, 3-OMe, 5-O^i^Pr	D-4	39#	0.05	0.16	5.84
**41**	CN	H	D-2	73#	0.21	0.22	5.28
**42**	CN	3-F	D-2	62#	0.08	0.11	5.42
**43**	Br	4-aza, 2,3-diOMe	D-2	27	0.03	0.06	5.61
**44**	Br	H	E-6	26	0.47	0.14	6.53
**45**	Br	H	E-4	44	0.53	0.25	6.53
**46**	CN	H	E-4	38#	0.08	0.2	5.18
**47**	Br	H	E-2	47	0.27	0.15	6.53
**48**	Br	H	F-6	39	0.11	0.11	6.50
**49**	CN	H	F-6	56#	0.37	0.36	5.18
**50**	Br	H	F-4	19	<0.02	0.06	6.50
**51**	Br	H	G	54	0.15	0.07	6.13
**52**	CN	H	G	83#	0.72	0.77	4.77
**53**	Br	4-aza, 2,3-diOMe	G	79	0.14	0.08	5.10
**54**	CN	4-aza, 2,3-diOMe	G	68#	0.58	0.58	3.75
**55**	Br	3-Me	H	42	0.04	0.08	6.54
**56**	CN	3-Me	H	70#	0.23	0.16	5.18
**57**	Br	3-Me	I	50	0.13	0.15	6.71
**58**	Br	4-aza, 2,3-diOMe	J	28	1.3	1.8	4.33
**59**	Br	4-aza, 2,3-diOMe	K	29	1.0	1.9	4.32
**60**	Br	4-aza, 2,3-diOMe	L	86	>2.5	>2.5	4.97
**61**	CN	4-aza, 2,3-diOMe	L	65#	4.6	4.5	3.61
**62**	Br	H	M	59	0.12	0.23	5.14
**63**	Br	4-aza, 2,3-diOMe	M	75	0.07	0.12	4.11
**64**	Br	4-aza, 2,3-diOMe	N	57	2.3	3.3	4.55

*Footnotes for Table 1*:^a^Yield in the final AB/CD coupling step for the 6-Br compounds, or (#) for the cyanation step for the 6-CN compounds. ^b^MIC_90_ (µg/mL); minimum inhibitory concentration for 90% inhibition of growth of *M.tb* strain H37Rv, determined under aerobic (replicating; MABA) (Ref. 13) or non-replicating (LORA) (Ref. 14) conditions, determined at the Institute for Tuberculosis Research, University of Illinois at Chicago. ^c^clogP calculated by ChemDraw Ultra v12.0.2. (CambridgeSoft).

The A/B subunits were synthesised by directed *ortho* lithiation of the methoxyquinoline (**I**), followed by quenching with a suitably functionalised aldehyde (**II**) to give the benzylic alcohols (**III**); subsequent reduction to the corresponding dihydro adducts (**IV**) was performed using Et_3_SiH/TFA (Scheme 1). Alternatively, the A/B subunits were synthesised by a Suzuki reaction between the boronic acid (**V**) and suitably functionalised benzyl bromides (**VI**).

The Mannich bases (**III**) for Table 1 compounds **2**–**4** (class A), **5**–**6** (class B), **41**–**43** (class D-2), and class G (**51**–**54**) were in the literature or commercially available. The remainder were prepared from the requisite bicyclic acids (**I**) via the corresponding Weinreb amides (**II**).

Table 1 reports data on 63 analogs of **1** (Fig. 1) where the naphthalene C-unit has been replaced by a variety of bicyclic moieties, grouped in decreasing order of lipophilicity of the C-unit bicycle. The concentrations of these compounds needed for growth inhibition of cultures of *M.tb* (strain H37Rv) were determined as MIC_90_ values under both replicating (MABA assay^13^) and non-replicating (LORA assay^14^) conditions. Selected representative compounds (**7**, **9**, **14**, **16**–**18**, **22**, **23**, **30**, **31**, **37**–**43**, **52**–**57**, **62**) were evaluated for mammalian cell toxicity in Vero (African green monkey kidney) cells^15^; all had IC_50_s > 10 µg/mL except for **39** (4 µg/mL), **40** (8 µg/mL), and **41** (8.6 µg/mL). All measured analogs, including bedaquiline, showed very high binding to human plasma protein (>99.9%) with the exception of one compound, **50**, which showed 98.6% binding (Table S1).

The compounds in Table 1 cover a range of 4.4 clogP units; compare, for example, compounds **5** and **63**. Within each C unit subset there was also limited variation of substituents in the B subunit, and only Br and CN substituents on the A subunit.

Compounds **2**–**6** contain either a 5-linked tetrahydronaphthalene unit or a 7-linked benzo[*b*]thiophenes, and are the most lipophilic compounds; all showed *in vitro* activity against M. tb comparable to bedaquiline. Compounds **7**–**13** explore the 4-linked 2,3-dihydro-1H-indene unit, which still provides quite potent yet lipophilic compounds. The 7-linked 2,3-dihydrobenzofuran unit was explored with a larger number of compounds (**14**–**31**), including more polar unit B substituents and a number of Br/CN pairs. These covered a wide range of overall lipophilicity (from clogP 7.4 to 4.2). Compounds **32**–**43** have the 2,3-dihydrobenzofuran unit linked through other available positions (2-, 4-, 5- and 6-). There is less variation in lipophilicity within this set, which makes it easier to compare the effect of positioning to activity against *M.tb*. Overall, this effect does not seem large, although **33** and **37** are exceptions. Compounds **44**–**50** contain relatively lipophilic *N*-methylindoles and *N*-methylindolines, and show somewhat reduced potency than the comparable dihydrobenzofurans. Two 6-linked *N*-methylindolines (**48**, **49**) had similar activity to the other methylindolines but the 4-linked analog **50** was much more potent (cf with **37**). The 7-linked 2,3-dihydrobenzofurans (**51**–**54**) included the very polar compound **54** (clogP 3.75) which was less effective. The two 4-linked benzo[*d*][1,3]dioxoles (**55**, **56**) and the 5-linked 2,3-dihydrobenzo[*b*][1,4]dioxin (**57**) had relatively lipophilic substituents in the B-subunit and retained reasonable potency. However, the 7-linked [1,3]dioxolo[4,5-*b*]pyridine (**58**), furo[3,2-*b*]pyridine K (**59**) and 8-linked 2,3-dihydro-[1,4]dioxino[2,3-*b*]pyridines (**60**, **61**), with a polar unit B, were among the most polar compounds in the set, with clogP values between 3.6 and 5.0, and overall showed more modest anti-TB activity *in vitro*. Finally, of the 4-linked furo[3,2-*c*]pyridines (**62**, **63**) and the 7-linked furo[2,3-*c*]pyridine (**64**), the latter was very polar and had attenuated activity.

### 2.2 Structure-activity relationships

Across all of the compounds, which encompass a 4.4-fold range in clogP value, high potency (low MIC_90_) correlates positively with overall lipophilicity (Eq. (1)); a trend noted previously for this^10^ and other^15^ classes of *M.tb* inhibitors). This has been suggested^16^ to be due to the very lipophilic cell wall of Mycobacteria.

(1) $$\begin{array}{cc} & {\text{logMIC}}_{90}(\text{MABA})=-0.40(\pm 0.06)(\text{clogP})+1.39(\pm 0.38)\\ & \text{n}=64\text{,r}=0.63\text{,P}=<0.001\text{,}{\text{F}}_{2\text{,}61}=41 \end{array}$$

However, inspection of individual compounds shows MIC_90_ potency dropping off by more than predicted by Eq. (1) for compounds with clogP values below about 5 (e.g., compounds **23**, **52**, **58**–**61**). It is not clear if this is a result of less target engagement or less penetration into, and/or more efflux out of, the bacterium. Nevertheless, many compounds (e.g., **13**, **25**, **27**, **29**, **37**, **63**) were similarly potent and more hydrophilic than bedaquiline.

We have previously^10^ discussed the use of 6-substituents other than Br on the quinoline ring of bedaquiline, and showed that the polar but electron-withdrawing CN substituent provided a substantial decrease in drug lipophilicity (about 1.4 clogP units), at the expense of only a 2–3-fold increase in MIC values against *M.tb*. The inclusion of 19 pairs of compounds where the 6-Br group of **1** was exchanged for a 6-CN group allowed a further evaluation of this trend. For the 15 pairs where we had definitive MIC_90_ data for both members (Table 1), the average increase in MABA and LORA MIC_90_s were similar, being 2.76- and 2.81-fold respectively.

Using a dummy variable for the presence of the CN substituent shows that the CN group provides, on average, a further small (0.18 log) reduction in MIC_90_, after the effect of its lower lipophilicity is allowed for.

(2) $$\begin{array}{cc} & {\text{logMIC}}_{90}(\text{MABA})=-0.45(\pm 0.08)(\text{clogP})-0.18(\pm 0.16)\text{X}+1.66(\pm 0.50)\\ & \text{n}=64\text{,r}=0.61\text{,}{\text{F}}_{3\text{,}62}=18\text{,where X}=1.0\;\text{for CN,}0.0\;\text{for Br} \end{array}$$

Note that in Eqs. (1), (2) the absolute values for the logMIC(MABA) data in Table 1 were used; removal of the < and > qualified data points from the calculation did not change the nature of the result.

A representative subset of the compounds of Table 1 were also evaluated for a number of pharmacological properties, and compared against bedaquiline (**1**) (Table 2). For the compounds with measured values, all except for **18**, **34**, and **36** had IC_50_’s > 10 µM for inhibition of the common CYP 3A4 oxidative metabolizing enzyme, and all showed no toxicity toward Vero green monkey kidney cells^15^ at a concentration of 10 µg/mL (data not shown). Bedaquiline itself (**1**) is a potent inhibitor of the hERG potassium channel (IC_50_ 1.6 µM in our assay, but also reported as 0.37 µM),^16^ which is seen as a potential cardiovascular liability, and has prompted comments that its use should be confined to settings where carefully selected patients can be closely monitored, and that combination with other QTc-prolonging drugs should be avoided.^17^ The IC_50_ for inhibition of hERG current for bedaquiline, measured in the patch clamp assay we utilized for this study, was 1.6 µM, which is in the same range as the published value. Compared to this, a few of the analogs (**5**, **24**, **28**, **30**) showed a significant (5–8-fold) improvement in this parameter, but there was no common feature to distinguish them from others which were much more potent hERG inhibitors (e.g., **14**, **16**, **34**, **36**, **55**, **56**). All of the compounds except **24**, **26**–**28** (which were four of the most lipophilic compounds) had significantly faster clearance rates in human liver microsomes (HCl_int_) and concomitantly shorter half-lives (Ht½) than did **1** (Table 2), which is in line with previous SAR observations.^11^ The same trend was observed across the whole set of compounds for which defined human liver microsome clearance data was obtained, with a modest correlation seen between lower lipophilicity and faster clearance.

(3) $$\begin{array}{cc} & \text{log}({\text{HCl}}_{\text{int}})=-0.26(\text{clogP})+2.60\\ & \text{n}=28\text{,r}=0.52\text{,P}=<0.0001\text{,F}=9.54 \end{array}$$

**Table 2:** Biochemical profiling of selected representative compounds of Table 1.

	IC_50_ (µM)					Log CFU redn			
No	CYP 3A4^a^	hERG^b^	HCl_int_^c^	Ht½^d^	F (%)^e^	20 mpk^f^	BDQ 10 mpk^g^	BDQ 20 mpk^h^	clogP^i^
**1**	>50	1.6	3	231	56		3.3–5.5	4.5–6.2	7.25
**5**	ND^j^	12	21	33	33	4.6	4.6	4.6	7.11
**7**	>10	3.8	13	52	34	5.7	4.5	5.6	7.09
**10**	ND	1-3^j^	31	23	25	ND	ND	ND	4.99
**11**	ND	∼3	9	75	51	ND	ND	ND	7.09
**14**	>10	0.8	14	50	52	3.3	4.3	4	5.28
**16**	>10	0.6	11	64	42	>5.7	4.1	5.7	6.64
**18**	7.8	ND	20	35	13	ND	ND	ND	5.56
**21**	ND	1-3^j^	16	42	ND	ND	ND	ND	6.48
**22**	>10	0.9	18	39	37	0.9	4.1	5.7	5.61
**24**	>10	>10	3	248	49	4.2	4.3	5.3	7.41
**25**	ND	3.3	65	11	21	ND	ND	ND	6.06
**26**	ND	7.5	<2.3	>300	73	5.6	3.3	4.5	6.87
**27**	ND	<1	3	277	ND	ND	ND	ND	5.53
**28**	ND	>10	<2.3	>300	41	5.4	3.3	4.5	7.19
**29**	ND	>2	4.9	141	25	5.3	5	6.1	5.84
**30**	ND	>10	5	133	46	ND	ND	ND	7.42
**34**	9.7	0.4	34	20	42	3.4	4.3	5.3	6.64
**36**	6.9	0.6	18	38	52	5.2	4.3	5.3	5.61
**37**	ND	1-3^j^	12	59	ND	ND	ND	ND	5.61
**42**	>10	0.9	43	16	33	3.7	4.1	5.7	5.42
**43**	>10	0.8	30	23	50	3.8	4.1	5.7	5.61
**48**	ND	<1	4.6	151	ND	ND	ND	ND	6.50
**50**	ND	1.3	12	56	35	ND	ND	ND	6.50
**54**	>10	>1	23	31	21	ND	ND	ND	3.75
**55**	>10	0.5	7	99	28	0.8	3.9	4.9	6.54
**56**	>10	0.3	15	47	38	0.2	5.5	6.2	5.18
**57**	>10	ND	4	182	ND	ND	ND	ND	6.71
**60**	ND	>3	8	91	61	ND	ND	ND	4.97

*Footnotes for Table 2*: ^a^Inhibition of CYP3A4 (human liver microsomes, 20 min exposure); ^b^Inhibition of the hERG channel (5-point manual patch-clamp assay, conducted at WuXi); ^c^Clearance (µL/min/mg) in human liver microsomes at 60 min (1 µM concentration); ^d^half-life (min) in human liver microsomes (1 µM concentration); ^e^Oral bioavailability (in male CD-1 mice, when given at 10 mg/kg); ^f,g,h^Log reduction of colony-forming units from the lungs of BALB/c mice compared to the untreated (vehicle only) control after daily oral dosing at 20^f^ mg/kg/day for each test compound, compared to 10^g^ and 20^h^ mg/kg/day bedaquiline tested in the same assay, with 12 continuous days of once per day dosing. ^i^clogP calculated by ChemDraw Ultra v12.0.2. (CambridgeSoft); ^j^ND: not done.

For the compounds profiled in Table 2, half-lives on incubation with human liver microsomes were extended by nearly 5-fold on average in the presence of the microsome inhibitor ketoconazole (Supplementary Data; Table S1), suggesting that the majority of the metabolism comes from CYP3A4, as for bedaquiline.

While these compounds were also similar to bedaquiline in their low aqueous solubility and high plasma protein binding (Supplementary Data; Table S1), most had acceptable bioavailability (F > 25% in mice). Finally, as shown previously,^10^, ^11^ the oral bioavailability of the compounds in mice was broadly inversely correlated with compound lipophilicity.

Several of the compounds were further evaluated for efficacy *in vivo* against acute murine TB. For these assessments, female BALB/c mice, infected via aerosol with *M. tuberculosis* Erdman,^18^ were treated by oral gavage with a dose of 20 mg/kg once daily for 12 continuous days, beginning on day 11 post-infection. Compounds were administered as a solution in 20% hydroxypropyl-beta-cyclodextrin, in water, adjusted to pH 3. Bedaquiline, dosed orally once daily at either 10 mg/kg or at 20 mg/kg, was administered as a comparator, and vehicle-treated mice were evaluated as a negative control. Mice were sacrificed on day 25, and the numbers of colony forming units (CFU) in the lungs were determined by plating on agar containing charcoal (to absorb compound and prevent drug carryover effects) and compared with the CFUs for bedaquiline and vehicle alone-treated mice. Of the compounds evaluated, the most effective analogs, where 20 mg/kg/day effected a reduction in lung CFU at least as great as that demonstrated by 10 mg/kg/day bedaquiline, tested alongside, (i.e., **5**, **7**, **16**, **24**, **26**, **28**, **29**, **36**) had lipophilicities (clogPs) ranging from 7.41 to 5.61, including two with CN-substituted A units, again suggesting the viability of a CN substituent to lower overall drug lipophilicity without compromising *in vivo* efficacy.

## 3 Conclusions

This work, part of a programme seeking improved analogs of bedaquiline (**1**), sought to evaluate the effects of replacing the naphthalene C-unit with a variety of bicyclic moieties of widely differing structures and lipophilicities. The results, which show an MIC_90_/lipophilicity relationship (Eq. (1)) broadly similar to that seen for other series of TB drugs, suggest that changes in the C-subunit are well-tolerated for this series. Encouragingly, many analogs with lower clogP values than bedaquiline were at least as potent against *M.tb*. Further, where tested, most analogs with at least similar potency to bedaquiline against *M.tb* had higher clearance in human liver microsomes, suggesting they may show shorter terminal half-lives than bedaquiline *in vivo*, with less risk of tissue over-accumulation. Among the analogs, there was a significant range in potency for hERG channel block (IC_50_’s from 0.4 to >10 µM). While there was no discernible SAR pattern to this, it is encouraging that such improvements in hERG liability can be seen for what is still a lipophilic aromatic strong base, and that anti-mycobacterial potency is still maintained when hERG inhibition is reduced. The four best compounds in his regard (**24**, **26**, **28**, **30**) were all of one class (D-7; 7-benzofuran), suggest that exploration of further C-unit variations might be of interest in this regard. The value of the A-unit CN substituent is also confirmed for its ability to lower overall drug lipophilicity (thus contributing to desirable physiochemical properties) without compromising *in vivo* efficacy.

## 4 Experimental

### 4.1 Chemistry

Final products were analysed by reverse-phase HPLC (Alltima C18 5 µm column, 150 × 3.2 mm; Alltech Associated, Inc., Deerfield, IL) using an Agilent HP1100 equipped with a diode-array detector. Mobile phases were gradients of 80% CH_3_CN/20% H_2_O (v/v) in 45 mM NH_4_HCO_2_ at pH 3.5 and 0.5 mL/min. Purity was determined by monitoring at 330 ± 50 nm and was ≥95% for all final products. Melting points were determined on an Electrothermal 9100 melting point apparatus. NMR spectra were obtained on a Bruker Avance 400 spectrometer at 400 MHz for ^1^H. Low-resolution atmospheric pressure chemical ionization (APCI) mass spectra were measured for organic solutions on a ThermoFinnigan Surveyor MSQ mass spectrometer, connected to a Gilson autosampler.

#### 4.1.1 Scheme 2. Synthesis of A/B units

##### 4.1.1.1 6-Bromo-3-((2,3-dihydrobenzo[*b*][1,4]dioxin-5-yl)methyl)-2-methoxyquinoline (**VI**: Y = 2,3-O(CH_2_)_2_O–)

To a solution of 2,3-dihydrobenzo[*b*][1,4]dioxine-5-carboxylic acid (5.00 g, 28.0 mmol) in THF (150 mL) at 0 °C was added lithium aluminium hydride (2.13 g, 56.0 mmol) in small portions. The reaction mixture was stirred at 0 °C for 10 min and stirred for a further 18 h at 20 °C. Water (150 mL) was added to the reaction mixture which was extracted with EtOAc (2 × 100 mL). The combined organic layers were washed with brine (100 mL), dried over Na_2_SO_4_, filtered and concentrated under reduced pressure to obtain (2,3-dihydrobenzo[*b*][1,4]dioxin-5-yl)methanol as a yellow oil (3.22 g, 99%). ^1^H NMR (CDCl_3_, 400 MHz) *δ* 6.87–6.79 (m, 3H), 4.66 (s, 2H), 4.32–4.30 (m, 2H), 4.28–4.25 (m, 2H), 2.19 (bs, 1H). Found: [M+H-18] = 149.5.

A solution of the above alcohol (3.75 g, 32.3 mmol) in Et_2_O (80 mL) was cooled to 0 °C and phosphorous tribromide (3.67 mL, 38.8 mmol) was added dropwise. The solution was stirred at 0 °C for 10 min, then at 20 °C for 1 h. Water (10 mL) was added cautiously to quench the excess of reagent and the mixture was diluted with diethyl ether and washed with water (3 × 50 mL). The combined organic layers were washed with brine (100 mL), dried over Na_2_SO_4_, filtered and concentrated under reduced pressure to obtain 5-(bromomethyl)-2,3-dihydrobenzo[*b*][1,4]dioxine as a brown solid (4.61 g, 62%). ^1^H NMR (CDCl_3_, 400 MHz) *δ* 6.91–6.77 (m, 3H), 4.52 (s, 2H), 4.35–4.33 (m, 2H), 4.29–4.27 (m, 2H). Found: [M+H-Br] = 149.5.

A mixture of the above bromide (4.6 g, 20.0 mmol), (6-bromo-2-methoxyquinolin-3-yl)boronic acid (**V**) (4.42 g, 15.4 mmol) and Cs_2_CO_3_ (11.54 g, 0.77 mmol) in toluene:DMF (60 mL, 2:1) was degassed under N_2_, then Pd(PPh_3_)_4_ (0.890 g, 0.77 mmol) was added, and the mixture heated at 110 °C for 4 h. The mixture was cooled to 20 °C, filtered through a plug of Celite, water (150 mL) was added and extracted with EtOAc (3 × 100 mL). The combined organic layers were washed with brine (100 mL), dried over Na_2_SO_4_, filtered and concentrated under reduced pressure to obtain a yellow residue. Purification by flash column chromatography using hexanes:EtOAc (9:1) gave 6-bromo-3-((2,3-dihydrobenzo[*b*][1,4]dioxin-5-yl)methyl)-2-methoxyquinoline (**VI**: Y = 2,3-O(CH_2_)_2_O–) as white solid (3.1 g, 52%). ^1^H NMR (CDCl_3_) *δ* 7.75 (d, *J* = 2.2 Hz, 1H), 7.73–7.70 (m, 1H), 7.61–7.59 (m, 1H), 7.43 (s, 1H), 6.84–6.78 (m, 2H), 6.72–6.70 (m, 1H), 4.27–4.22 (m, 4H), 4.10 (s, 3H), 3.98 (s, 2H). Found: [M+H] = 386.6.

##### 4.1.1.2 6-Bromo-3-((2,6-diethoxypyridin-4-yl)methyl)-2-methoxyquinoline (**VI**: Y = 4-aza, 3,5 diOEt)

Trimethylborate (3.88 mL, 34.2 mmol) and borane-dimethylsulfide (3.25 mL, 34.3 mmol) were added sequentially to a solution of 2,6-diethoxyisonicotinic acid (2.41 g, 11.4 mmol) in THF (100 mL, dist. Na) at 0 °C and the mixture was stirred at 20 °C for 18 h. The solution was cooled to 0 °C and methanol was cautiously added to quench excess borane. Removal of the solvent gave a solid which was partitioned between EtOAc and water, the organic fraction was dried and evaporated. Column chromatography (3:1 hexanes:EtOAc) gave 2,6-diethoxypyridin-4-yl)methanol (2.13 g, 95%). M.p. 54–56 °C. ^1^H NMR (DMSO‑*d*_6_) *δ* 6.27 (d, *J* = 0.6 Hz, 2H), 4.62 (d, *J* = 6.2 Hz, 2H), 4.31 (q, *J* = 7.1 Hz, 4H), 1.72 (t, *J* = 6.2 Hz, 1H), 1.38 (t, *J* = 7.1 Hz, 6H). Found: [M+H] = 198.1.

A solution of the above alcohol (2.07 g, 10.5 mmol) in DCM (100 mL, anhydrous) at 0 °C was treated sequentially with Et_3_N (2.93 mL, 21.0 mmol) and mesyl chloride (1.22 mL, 15.8 mmol), the mixture was stirred at 0 °C for 1 h then partitioned between DCM and water. The organic fraction was dried and evaporated and the residue was dissolved in acetone (100 mL), LiBr (9.15 g, 105 mmol) was added and the mixture was refluxed for 1 h then evaporated. The residue was partitioned between DCM and water and the organic fraction was dried and evaporated. Column chromatography (DCM) gave 4-(bromomethyl)-2,6-diethoxypyridine (2.63 g, 92%), mp 37–39 °C. ^1^H NMR (CDCl_3_) *δ* 6.28 (s, 2H), 4.31 (q, *J* = 7.1 Hz, 4H), 4.28 (s, 2H), 1.38 (t, *J* = 7.1 Hz, 6H). Found: [M+H] = 260.5.

A mixture of the above bromide (2.20 g, 7.80 mmol), 4-(bromomethyl)-2,6-diethoxypyridine (2.13 g, 8.20 mmol) and Cs_2_CO_3_ (5.13 g, 15.6 mmol) in toluene (40 mL) and DMF (20 mL) was purged with nitrogen. Pd(PPh_3_)_4_ (0.18 g, 0.156 mmol) was added, the mixture was purged with nitrogen, then heated to 80 °C under nitrogen for 3 h. The reaction was partitioned between EtOAc and water and the organic fraction was dried and evaporated. Column chromatography with 3:1 hexanes:DCM eluted non polar impurities, elution with 1:1 hexanes:DCM gave 6-bromo-3-((2,6-diethoxypyridin-4-yl)methyl)-2-methoxyquinoline (**VI**: Y = 4-aza, 3.5 diOEt) (2.35 g, 72%), mp 91–92 °C. ^1^H NMR (CDCl_3_) *δ* 7.77 (d, *J* = 2.2 Hz, 1H), 7.69 (d, *J* = 8.0 Hz, 1H), 7.62 (dd, *J* = 8.8, 2.2 Hz, 1H), 7.58 (s, 1H), 6.13 (s, 2H), 4.29 (q, *J* = 7.1 Hz, 4H), 4.06 (s, 3H), 3.90 (s, 2H), 1.36 (t, *J* = 7.1 Hz, 6H). Found: [M+H] = ] = 417.1.

##### 4.1.1.3 6-Bromo-3-((2-isopropoxy-6-methoxypyridin-4-yl)methyl)-2-methoxyquinoline (**VI**: Y = 4-aza, 3-OMe, 5-O*^i^*Pr)

A suspension of 2,6-dihydroxyisonicotinic acid (10.00 g, 64.5 mmol) in MeOH (60 mL) was treated dropwise with H_2_SO_4_ (10 mL, 18.4 M, 184 mmol). The solution was refluxed for 72 h and then evaporated. The residue was treated with sat. aq. NaHCO_3_ to pH 8 and extracted with EtOAc (3 × 200 mL). The organic extracts were washed with sat. aq. NaHCO_3_ and brine, then dried and evaporated to give methyl 2-hydroxy-6-methoxyisonicotinate (3.55 g, 30%) as a tan solid, mp 183–185 °C. ^1^H NMR (DMSO‑*d*_6_) *δ* 11.2 (bs, 1H), 6.61 (bs, 2H), 3.84 (s, 3H), 3.83 (s, 3H). Found: [M+H] = 184.2.

A solution of the above alcohol (5.04 g, 27.5 mmol) in DMF (100 mL, anhydrous) was treated with K_2_CO_3_ (4.75 g, 34.4 mmol) and then 2-iodopropane (3.43 mL, 34.4 mmol). The mixture was stirred at 20 °C for 24 h, more 2-iodopropane (3.43 mL, 34.3 mmol) was added and the mixture was stirred for a further 72 h then partitioned between EtOAc and water and the aqueous layer was extracted further with EtOAc. The organic fractions were washed with water, dried and evaporated. Chromatography (DCM) gave methyl 2-isopropoxy-6-methoxyisonicotinate (6.21 g, 100%) as a colourless oil. ^1^H NMR (CDCl_3_) *δ* 6.81 (s, 2H), 5.24 (sp, *J* = 6.2 Hz, 1H), 3.92 (s, 3H), 3.90 (s, 3H), 1.36 (d, *J* = 6.2 Hz, 6H). Found: [M+H] = 226.2.

A solution of LiOH (1.98 g, 82.7 mmol) in water (60 mL) was added to a solution of the above ester (6.20 g, 27.5 mmol) in MeOH (60 mL) and THF (60 mL) and the solution was stirred at 20 °C for 18 h and then evaporated. The residue was dissolved in water (150 mL) and acidified to pH 3 with 2 M HCl. The precipitate was filtered and dried to give 2-isopropoxy-6-methoxyisonicotinic acid (5.33 g, 92%) as a white solid, mp 120–121 °C. ^1^H NMR (DMSO‑*d*_6_) *δ* 13.50 (bs, 1H), 6.70 (d, *J* = 1.0 Hz, 1H), 6.66 (d, *J* = 1.0 Hz, 1H), 5.20 (sp, *J* = 6.2 Hz, 1H), 3.86 (s, 3H), 1.31 (d, *J* = 6.2 Hz, 6H). Found: [M+H] = 212.1.

Trimethylborate (6.81 mL, 59.6 mmol) and borane dimethylsulfide complex (5.66 mL, 59.7 mmol) were added sequentially to a solution of the above acid (6.30 g, 29.8 mmol) in THF (100 mL, dist. Na) at 0 °C, the mixture was stirred at 20 °C for 18 h. The solution was cooled to 0 °C and quenched with methanol. Removal of the solvent gave a white solid, which was partitioned between EtOAc and water, the organic fraction was dried and evaporated to give 2-isopropoxy-6-methoxypyridin-4-yl)methanol (5.86 g, 99%) as white solid, mp 121–122 °C. ^1^H NMR (CDCl_3_) *δ* 6.26 (t, *J* = 0.8 Hz, 1H), 6.25 (t, *J* = 0.8 Hz, 1H), 5.23 (sp, *J* = 6.2 Hz, 1H), 4.61 (d, *J* = 6.2 Hz, 2H), 3.88 (s, 3H), 1.73 (t, *J* = 6.2 Hz, 1H), 1.34 (d, *J* = 6.2 Hz, 6H). Found: [M+H] = 212.2 (M−OH+MeO).

A solution of the above alcohol (5.86 g, 29.9 mmol) in DCM (100 mL, anhydrous) at 0 °C was treated with Et_3_N (8.3 mL, 59.5 mmol) and then MsCl (3.47 mL, 44.8 mmol), the mixture was stirred at 0 °C for 1 h then partitioned between DCM and water. The organic fraction was dried and evaporated and the residue was dissolved in acetone (200 mL), LiBr (25.9 g, 299 mmol) was added and the mixture was refluxed for 1 h then evaporated. The residue was partitioned between DCM and water; the organic fraction was dried and evaporated. Column chromatography (DCM) gave 4-(bromomethyl)-2-isopropoxy-6-methoxypyridine (6.98 g, 90%) as a colourless oil. ^1^H NMR (CDCl_3_) *δ* 6.28 (s, 1H), 6.27 (s, 1H), 5.23 (sp, *J* = 6.2 Hz, 1H), 4.27 (s, 2H), 3.88 (s, 3H), 1.35 (d, *J* = 6.3 Hz, 6H). Found: [M+H] = 260.1.

A mixture of the above bromide (7.45 g, 26.4 mmol), 4-(bromomethyl)-2-isopropoxy-6-methoxypyridine (6.84 g, 26.4 mmol) and Cs_2_CO_3_ (17.3 g, 52.7 mmol) in toluene (100 mL) and DMF (50 mL) was purged with nitrogen. Pd(PPh_3_)_4_ (0.61 g, 0.528 mmol) was added, the mixture was purged with nitrogen and then heated to 80 °C under nitrogen for 3 h. The reaction mixture was partitioned between EtOAc and water and the organic fraction was dried and evaporated. Column chromatography with 3:1 hexanes:DCM eluted impurities, then elution with 1:1 hexanes:DCM gave 6-bromo-3-((2-isopropoxy-6-methoxypyridin-4-yl)methyl)-2-methoxyquinoline (**VI**: Y = 4-aza, 3-OMe, 5-O*^i^*Pr) (8.59 g, 78%) as a white solid, mp 90–91 °C. ^1^H NMR (CDCl_3_) *δ* 7.78 (d, *J* = 2.1 Hz, 1H), 7.69 (d, *J* = 8.9 Hz, 1H), 7.62 (dd, *J* = 8.9, 2.2 Hz, 1H), 7.58 (s, 1H), 6.14 (d, *J* = 0.9 Hz, 1H), 6.10 (d, *J* = 0.9 Hz, 1H), 5.22 (sp, *J* = 6.2 Hz, 1H), 4.06 (s, 3H), 3.90 (s, 2H), 3.87 (s, 3H), 1.33 (d, *J* = 6.2 Hz, 6H). Found: [M+H] = 417.1.

##### 4.1.1.4 6-Bromo-3-((2-ethoxy-6-methoxypyridin-4-yl)methyl)-2-methoxyquinoline (**VI**: Y = 4-aza, 3-OMe, 5-OEt)

A solution of methyl 2-hydroxy-6-methoxyisonicotinate (6.96 g, 38.0 mmol) in DMF (100 mL, anhydrous) was treated with K_2_CO_3_ (6.57 g, 47.6 mmol) and then iodoethane (3.85 mL, 47.6 mmol). The mixture was stirred at 20 °C for 24 h, partitioned between EtOAc and water and the aqueous layer was extracted with EtOAc. The organic fractions were washed with water, dried and evaporated, chromatography (DCM) gave methyl 2-ethoxy-6-methoxyisonicotinate (6.20 g, 77%) as a white solid, mp 38–431 °C. ^1^H NMR (CDCl_3_) *δ* 6.84 (s, 2H), 4.35 (q, *J* = 7.1 Hz, 2H), 3.93 (s, 3H), 3.91 (s, 3H), 1.40 (t, *J* = 7.1 Hz, 3H). Found: [M+H] = 212.1.

A solution of LiOH (2.10 g, 87.7 mmol) in water (60 mL) was added to a solution of the above ester (6.20 g, 29.4 mmol) in MeOH (60 mL) and THF (60 mL), the solution was stirred at 20 °C for 18 h and then evaporated. The residue was dissolved in water (150 mL) and acidified to pH 3 with 2 M HCl. The precipitate was filtered and dried to give 2-ethoxy-6-methoxyisonicotinic acid (5.61 g, 97%) as a white solid, mp 137–139 °C. ^1^H NMR (DMSO‑*d*_6_) *δ* 13.54 (bs, 1H), 6.73 (d, *J* = 1.0 Hz, 1H), 6.71 (d, *J* = 1.0 Hz, 1H), 4.32 (q, *J* = 7.0 Hz, 2H), 3.87 (s, 3H), 1.33 (t, *J* = 7.0 Hz, 3H). Found: [M+H] = 198.2.

Trimethylborate (6.06 mL, 53.1 mmol) and then borane-dimethylsulfide (5.04 mL, 53.1 mmol) were added to a solution of the above acid (5.24 g, 26.6 mmol) in THF (100 mL, dist. Na) at 0 °C and the mixture was stirred at 20 °C for 18 h. The solution was cooled to 0 °C and quenched with methanol. Removal of the solvent gave a solid which was partitioned between EtOAc and water, the organic fraction was dried and evaporated to give (2-ethoxy-6-methoxypyridin-4-yl)methanol (4.79 g, 98%), mp 68–71 °C. ^1^H NMR (CDCl_3_) *δ* 6.29 (bd, *J* = 0.7 Hz, 1H), 6.28 (bd, *J* = 0.7 Hz, 1H), 4.63 (d, *J* = 6.2 Hz, 2H), 4.32 (q, *J* = 7.1 Hz, 2H), 3.90 (s, 3H), 1.76 (t, *J* = 6.2 Hz, 1H), 1.39 (t, *J* = 7.1 Hz, 3H). Found: [M+H] =  184.2.

A solution of the above alcohol (4.72 g, 25.9 mmol) in DCM (100 mL, anhydrous) at 0 °C was treated sequentially with Et_3_N (7.22 mL, 51.8 mmol) and mesyl chloride (3.00 mL, 38.8 mmol), the mixture was stirred at 0 °C for 1 h then partitioned between DCM and water. The organic fraction was dried and evaporated and the residue was dissolved in acetone (200 mL), LiBr (22.5 g, 259 mmol) was added and the mixture was refluxed for 1 h then evaporated. The residue was partitioned between DCM and water; the organic fraction was dried and evaporated. Column chromatography (DCM) gave 4-(bromomethyl)-2-ethoxy-6-methoxypyridine (5.78 g, 91%) as a white solid, mp 41–42 °C. ^1^H NMR (CDCl_3_) *δ* 6.30 (s, 2H), 4.33 (q, *J* = 7.1 Hz, 2H), 4.28 (s, 2H), 3.90 (s, 3H), 1.39 (t, *J* = 7.1 Hz, 3H). Found: [M+H] = 246.0.

A mixture of the above acid (5.77 g, 23.4 mmol), (6-bromo-2-methoxyquinolin-3-yl)boronic acid (**V**; 6.61 g, 23.4 mmol) and Cs_2_CO_3_ (15.25 g, 46.5 mmol) in toluene (100 mL) and DMF (50 mL) was purged with nitrogen. Pd(PPh_3_)_4_ (0.54 g, 0.465 mmol) was added, the mixture was purged with nitrogen then heated to 80 °C under nitrogen for 3 h. The reaction mixture was partitioned between EtOAc and water and the organic fraction was dried and evaporated. Column chromatography with 3:1 hexanes:DCM eluted impurities, then elution with 1:1 hexanes:DCM gave 6-bromo-3-((2-ethoxy-6-methoxypyridin-4-yl)methyl)-2-methoxyquinoline (**VI**: Y = 4-aza, 3-OMe, 5-OEt) (6.69 g, 71%) as a white solid, mp 104–106 °C. ^1^H NMR (CDCl_3_) *δ* 7.78 (d, *J* = 2.1 Hz, 1H), 7.69 (d, *J* = 8.9 Hz, 1H), 7.63 (dd, *J* = 8.9, 2.2 Hz, 1H), 7.57 (s, 1H), 6.15 (d, *J* = 8.0 Hz, 2H), 4.31 (q, *J* = 7.1 Hz, 2H), 4.06 (s, 3H), 3.91 (s, 2H), 3.88 (s, 3H), 1.37 (t, *J* = 7.1 Hz, 3H). Found: [M+H] = 403.1.

##### 4.1.1.5 6-Bromo-2-methoxy-3-((2-methoxy-6-propoxypyridin-4-yl)methyl)quinoline (**VI**: Y = 4-aza, 3-OMe, 5-O*^n^*Pr)

A solution of methyl 2-hydroxy-6-methoxyisonicotinate (6.00 g, 32.8 mmol) in DMF (100 mL, anhydrous) was treated with K_2_CO_3_ (6.80 g, 49.2 mmol) and then 1-iodopropane (4.8 mL, 49.2 mmol). The mixture was stirred at 20 °C for 48 h, partitioned between EtOAc and water and the aqueous layer was extracted with EtOAc. The organic fractions were washed with water, dried and evaporated. Column chromatography (DCM) gave methyl 2-methoxy-6-propoxyisonicotinate (6.59 g, 89%) as a colourless oil. ^1^H NMR (CDCl_3_) *δ* 6.85 (d, *J* = 1.0 Hz, 1H), 6.83 (d, *J* = 1.0 Hz, 1H), 4.24 (t, *J* = 6.7 Hz, 2H), 3.93 (s, 3H), 3.91 (s, 3H), 1.80 (qt, *J* = 7.4, 6.7 Hz, 2H), 1.02 (t, *J* = 7.4 Hz, 3H). Found: [M+H] = 226.1.

A solution of LiOH (2.04 g, 85.2 mmol) in water (60 mL) was added to a solution of the above ester (6.40 g, 28.4 mmol) in THF (60 mL) and MeOH (60 mL), the solution was stirred at 20 °C for 18 h and then evaporated. The residue was dissolved in water (200 mL) and acidified to pH 3 with 2 M HCl. The precipitate was filtered and dried to give 2-methoxy-6-propoxyisonicotinic acid (5.39 g, 90%) as a white solid, mp 141–143 °C. ^1^H NMR (DMSO‑*d*_6_) *δ* 13.53 (bs, 1H), 6.73 (d, *J* = 1.0 Hz, 1H), 6.72 (d, *J* = 1.0 Hz, 1H), 4.23 (t, *J* = 6.6 Hz, 2H), 3.87 (s, 3H), 1.73 (qt, *J* = 7.4, 6.6 Hz, 2H), 0.96 (t, *J* = 7.4 Hz, 3H). Found: [M+H] = 212.1.

Trimethylborate (5.8 mL, 50.8 mmol) and borane dimethylsulfide complex (4.8 mL, 50.6 mmol) were added sequentially to a solution of the above acid (5.39 g, 25.5 mmol) in THF (100 mL, dist. Na) at 0 °C, and the mixture was stirred at 20 °C for 16 h. The solution was cooled to 0 °C and methanol was cautiously added to quench the reaction. Removal of the solvent gave a solid which was partitioned between EtOAc and water, the organic fraction was dried and evaporated. Column chromatography (3:1 hexanes:EtOAc) gave (2-methoxy-6-propoxypyridin-4-yl)methanol (5.04 g, 100%) as a colourless oil. ^1^H NMR (CDCl_3_) *δ* 6.28 (d, *J* = 0.8 Hz, 1H), 6.26 (d, *J* = 0.8 Hz, 1H), 4.62 (d, *J* = 6.2 Hz, 2H), 4.31 (q, *J* = 7.1 Hz, 2H), 4.20 (t, *J* = 6.8 Hz, 2H), 1.72–1.83 (m, 3H), 1.38 (t, *J* = 7.0 Hz, 3H), 1.00 (t, *J* = 7.4 Hz, 3H). Found: [M+H] = 198.2.

A solution of the above alcohol (5.00 g, 25.4 mmol) in DCM (100 mL, anhydrous) at 0 °C was treated with Et_3_N (7.07 mL, 50.7 mmol) and then mesyl chloride (2.94 mL, 38.0 mmol), the mixture was stirred at 0 °C for 1 h then partitioned between DCM and water. The organic fraction was dried and evaporated and the residue was dissolved in acetone (150 mL), LiBr (22.0 g, 253 mmol) was added and the mixture was refluxed for 1 h then evaporated. The residue was partitioned between DCM and water; the organic fraction was dried and evaporated. Column chromatography (DCM) gave 4-(bromomethyl)-2-methoxy-6-propoxypyridine (6.15 g, 93%) as a colourless oil. ^1^H NMR (CDCl_3_) *δ* 6.31 (d, *J* = 1.0 Hz, 1H), 6.30 (d, *J* = 1.0 Hz, 1H), 4.28 (s, 2H), 4.22 (t, *J* = 6.7 Hz, 2H), 3.90 (s, 3H), 1.79 (qt, *J* = 7.4, 6.7 Hz, 2H), 1.02 (t, *J* = 7.4 Hz, 3H). Found: [M+H] = 260.5.

A mixture of (6-bromo-2-methoxyquinolin-3-yl)boronic acid (**V**; 6.62 g, 23.5 mmol) and the above bromide (6.11 g, 23.5 mmol) and Cs_2_CO_3_ (15.3 g, 47.0 mmol) in toluene (66 mL) and DMF (33 mL) was purged with nitrogen. Pd(PPh_3_)_4_ (0.54 g, 0.47 mmol) was added, the mixture was purged with nitrogen then heated to 80 °C under nitrogen for 3 h. The reaction was partitioned between EtOAc and water and the organic fraction was dried and evaporated. Column chromatography with 1:1 DCM:hexanes gave an impure product which was rechromatographed using a gradient of 2:1 hexanes:DCM to1:1 hexanes:DCM to give 6-bromo-2-methoxy-3-((2-methoxy-6-propoxypyridin-4-yl)methyl)quinoline (**VI**: Y = 4-aza, 3-OMe, 5-O*^n^*Pr) (6.63 g, 68%) as a white solid, mp 68–71 °C. ^1^H NMR (CDCl_3_) *δ* 7.78 (d, *J* = 2.2 Hz, 1H), 7.69 (d, *J* = 8.9 Hz, 1H), 7.63 (dd, *J* = 8.9, 2.2 Hz, 1H), 7.58 (s, 1H), 6.15 (s, 1H), 6.14 (s, 1H), 4.21 (t, *J* = 6.8 Hz, 2H), 4.06 (s, 3H), 3.91 (s, 2H), 3.88 (s, 3H), 1.79 (qt, *J* = 7.4, 6.8 Hz, 2H), 1.00 (t, *J* = 7.4 Hz, 3H). Found: [M+H] = 417.1.

##### 4.1.1.6 6-Bromo-3-((2-ethoxy-6-propoxypyridin-4-yl)methyl)-2-methoxyquinoline(**VI**: Y = 4-aza, 3-OEt, 5-OPr)

A suspension of 2,6-dihydroxyisonicotinic acid (40.00 g, 258 mmol) in EtOH (300 mL) was treated dropwise with H_2_SO_4_ (40 mL, 18.4 M, 752 mmol). The solution was refluxed for 72 h and then evaporated. The residue was treated with sat. aq. NaHCO_3_ to pH 8 and extracted with EtOAc (3 × 200 mL). The organic extracts were washed with sat. aq. NaHCO_3_ and brine, then dried and evaporated to give ethyl 2-ethoxy-6-hydroxyisonicotinate (10.86 g, 20%) as a tan solid, mp 102–104 °C. ^1^H NMR (DMSO‑*d*_6_) *δ* 11.45 (bs, 1H), 6.59 (d, *J* = 1.0 Hz, 1H), 6.57 (s, 1H), 4.29 (q, *J* = 7.1 Hz, 2H), 4.25 (q, *J* = 7.0 Hz, 2H), 1.298 (t, *J* = 7.1 Hz, 3H), 1.296 (t, *J* = 7.0 Hz, 3H).

A solution of the above hydroxyester (10.0 g, 47.3 mmol) in DMF (100 mL, anhydrous) was treated with K_2_CO_3_ (5.8 g, 59.2 mmol) and then 2-iodopropane (8.17 mL, 59.4 mmol). The mixture was stirred at 20 °C for 72 h. The mixture was partitioned between EtOAc and water and the aqueous layer was extracted further with EtOAc. The organic fractions were washed with water, dried and evaporated. Chromatography (DCM) gave ethyl 2-ethoxy-6-propoxyisonicotinate (8.98 g, 75%) as a colourless oil. ^1^H NMR (CDCl_3_) *δ* 6.84 (d, *J* = 1.1 Hz, 1H), 6.83 (d, *J* = 1.1 Hz, 1H), 4.36 (q, *J* = 7.1 Hz, 2H), 4.32 (q, *J* = 7.1 Hz, 2H), 4.23 (t, *J* = 6.7 Hz, 2H), 1.80 (qt, *J* = 7.4, 6.8 Hz, 2H), 1.40 (t, *J* = 7.1 Hz, 3H), 1.37 (t, *J* = 7.1 Hz, 3H), 1.02 (t, *J* = 7.4 Hz, 3H).

A solution of LiOH (2.53 g, 106 mmol) in water (60 mL) was added to a solution of the above ester (8.96 g, 35.4 mmol) in MeOH (60 mL) and THF (60 mL), the solution was stirred at 20 °C for 18 h and then evaporated. The residue was dissolved in water (150 mL) and acidified to pH 3 with 2 M HCl. The precipitate was filtered and dried to give 2-ethoxy-6-propoxyisonicotinic acid (**S20**) (7.96 g, quant.) as a white solid. ^1^H NMR (DMSO‑*d*_6_) *δ* 13.52 (bs, 1H), 6.80 (d, *J* = 1.0 Hz, 1H), 6.70 (d, *J* = 1.0 Hz, 1H), 4.30 (q, *J* = 7.0 Hz, 2H), 4.20 (t, *J* = 6.6 Hz, 2H), 1.73 (qt, *J* = 7.4, 6.8 Hz, 2H), 1.32 (t, *J* = 7.0 Hz, 3H), 0.96 (t, *J* = 7.4 Hz, 3H).

Trimethylborate (5.3 mL, 46 mmol) and borane dimethylsulfide complex (4.4 mL, 46 mmol) were added sequentially to a solution of the above acid (5.23 g, 23.2 mmol) in THF (100 mL, dist. Na) at 0 °C, the mixture was stirred at 20 °C for 18 h. The solution was cooled to 0 °C and quenched with methanol. Removal of the solvent gave a white solid, which was partitioned between EtOAc and water, the organic fraction was dried and evaporated. Column chromatography (3:1 hexanes:EtOAc) gave (2-ethoxy-6-propoxypyridin-4-yl)methanol (4.764 g, 97%) as a white solid, mp 36–39 °C. ^1^H NMR (CDCl_3_) *δ* 6.28 (d, *J* = 0.8 Hz, 1H), 6.26 (d, *J* = 0.8 Hz, 1H), 4.61 (d, *J* = 6.2 Hz, 2H), 4.31 (q, *J* = 7.1 Hz, 2H), 4.20 (t, *J* = 6.8 Hz, 2H), 1.78 (qt, *J* = 7.4, 6.8 Hz, 2H), 1.74 (t, *J* = 6.2 Hz, 1H), 1.38 (t, *J* = 7.0 Hz, 3H), 1.00 (t, *J* = 7.4 Hz, 3H).

A solution of the above alcohol (4.73 g, 22.4 mmol) in DCM (100 mL, anhydrous) at 0 °C was treated with Et_3_N (6.24 mL, 44.8 mmol) and then MsCl (2.6 mL, 33.6 mmol), the mixture was stirred at 0 °C for 1 h then partitioned between DCM and water. The organic fraction was dried and evaporated and the residue was dissolved in acetone (200 mL), LiBr (19.5 g, 225 mmol) was added and the mixture was refluxed for 1 h then evaporated. The residue was partitioned between DCM and water; the organic fraction was dried and evaporated. Column chromatography (DCM) gave 4-(bromomethyl)-2-ethoxy-6-propoxypyridine (5.817 g, 95%) as a colourless oil. ^1^H NMR (CDCl_3_) *δ* 6.29 (d, *J* = 1.0 Hz, 1H), 6.27 (d, *J* = 1.0 Hz, 1H), 4.31 (q, *J* = 7.1 Hz, 2H), 4.28 (s, 2H), 4.20 (t, *J* = 6.7 Hz, 2H), 1.78 (qt, *J* = 7.4, 6.8 Hz, 2H), 1.38 (t, *J* = 7.0 Hz, 3H), 1.00 (t, *J* = 7.4 Hz, 3H).

A mixture of the above bromide (5.81 g, 21.2 mmol), (6-bromo-2-methoxyquinolin-3-yl)boronic acid (**V**, 5.98 g, 21.2 mmol), and Cs_2_CO_3_ (13.8 g, 42.4 mmol) in toluene (133 mL) and DMF (66 mL) was purged with nitrogen. Pd(PPh_3_)_4_ (0.49 g, 0.42 mmol) was added, the mixture was purged with nitrogen and then heated to 80 °C under nitrogen for 3 h. The reaction mixture was partitioned between EtOAc and water and the organic fraction was dried and evaporated. Column chromatography with 3:1 hexanes:DCM eluted impurities, then elution with 1:1 hexanes:DCM gave 6-bromo-3-((2-ethoxy-6-propoxypyridin-4-yl)methyl)-2-methoxyquinoline (**VI**: Y = 4-aza, 3-OEt, 5-OPr) (6.644 g, 73%) as a white solid, mp 69–71 °C. ^1^H NMR (CDCl_3_) *δ* 7.77 (d, *J* = 2.1 Hz, 1H), 7.69 (d, *J* = 8.9 Hz, 1H), 7.62 (dd, *J* = 8.9, 2.2 Hz, 1H), 7.57 (s, 1H), 6.14 (s, 1H), 6.12 (s, 1H), 4.29 (q, *J* = 7.1 Hz, 2H), 4.19 (t, *J* = 6.7 Hz, 2H), 4.06 (s, 3H), 3.90 (s, 2H), 1.77 (qt, *J* = 7.4, 6.7 Hz, 2H), 1.36 (t, *J* = 7.1 Hz, 3H), 1.00 (t, *J* = 7.4 Hz, 3H). Found: [M+H] = 433.21, 431.1.

#### 4.1.2 Scheme 3A: synthesis of Mannich base C/D units via Weinreb amides

##### 4.1.2.1 1-(2,3-Dihydro-1H-inden-4-yl)-3-(dimethylamino)propan-1-one (class C)

To a solution of 2,3-dihydro-1*H*-indene-4-carboxylic acid (4.40 g, 27.5 mmol) in DCM (250 mL) was added DMF (0.426 mL) followed by dropwise addition of oxalyl chloride (4.19 g, 33.0 mmol). The reaction mixture was stirred for 1 h, cooled to 0 °C, then *N*,*O*-dimethylhydroxylamine hydrochloride (2.95 g, 30.3 mmol) and pyridine (7.32 mL, 90.7 mmol) were added and the reaction was stirred at 20 °C for 18 h. The mixture was poured onto sat. aqueous NaHCO_3_ (150 mL) and extracted with DCM (3 × 100 mL). The combined organic layers were dried over Na_2_SO_4_, filtered and concentrated under reduced pressure to obtain crude *N*-methoxy-*N*-methyl-2,3-dihydro-1H-indene-4-carboxamide, which was used directly (5.64 g, 100%). ^1^H NMR (CDCl_3_) *δ* 7.21–7.13 (m, 3H), 3.56 (s, 3H), 3.31 (s, 3H), 2.98–2.91 (m, 4H), 2.11–2.04 (m, 2H). Found: [M+H] = 206.5.

**Scheme 3:**
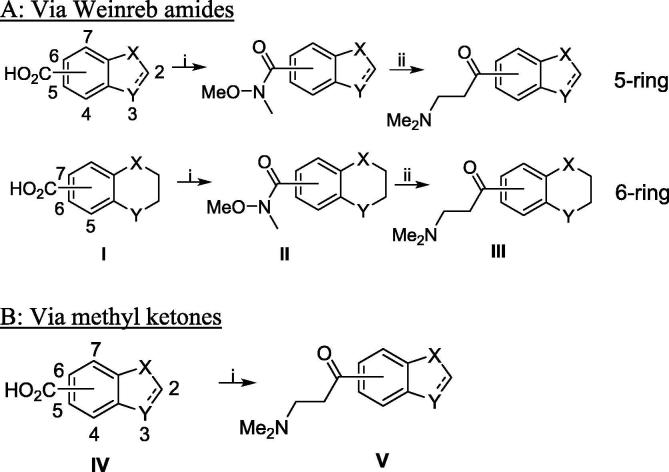
Synthesis of Mannich base C/D units.

A solution of the above crude amide (5.64 g, 27.5 mmol) in THF (150 mL) at 0 °C was treated with vinylmagnesium bromide (1M solution in THF, 57.7 mL, 57.7 mmol) and the solution was stirred for 3.5 h at 0 °C. Dimethylamine (2M solution in THF, 57.7 mL, 115.5 mmol) was added to the reaction mixture followed by water (60 mL). After 30 min stirring at 20 °C, the reaction mixture was concentrated under reduced pressure to obtain a brownish residue. This was extracted with EtOAc (3 × 200 mL). The combined organic layers were washed with brine (100 mL), dried over Na_2_SO_4_, filtered and concentrated under reduced pressure to obtain 1-(2,3-dihydro-1H-inden-4-yl)-3-(dimethylamino)propan-1-one as a brown oil (5.66 g, 95%). ^1^H NMR (CDCl_3_) *δ* 7.66 (d, *J* = 7.8 Hz, 1H), 7.34 (d, *J* = 7.4 Hz, 1H), 7.20 (t, *J* = 7.6 Hz, 1H), 3.24 (t, *J* = 7.5 Hz, 2H), 3.11 (t, *J* = 7.3 Hz, 2H), 2.88 (t, *J* = 7.5 Hz, 2H), 2.72 (t, *J* = 7.3 Hz, 2H), 2.27 (s, 6H), 2.04 (t, *J* = 7.5 Hz, 2H). Found: [M+H] = 218.6.

##### 4.1.2.2 1-(Benzofuran-2-yl)-3-(dimethylamino)propan-1-one (class D-2)

Oxalyl chloride (3.13 mL, 3.70 mmol) was added to a suspension of benzofuran-2-carboxylic acid (5.00 g, 3.08 mmol) in DCM (100 mL, anhydrous) and DMF (0.1 mL, 1.3 mmol) at 20 °C. The mixture was stirred at 20 °C for 1 h to give a colourless solution which was cooled to 0 °C. *N*,*O*-Dimethylhydroxylamine hydrochloride (3.31 g, 3.39 mmol) and pyridine (7.5 mL, 9.27 mmol) were added sequentially and the mixture was stirred at 20 °C for 18 h, then partitioned between EtOAc and sat. aq. NaHCO_3_. Column chromatography (3:1 hexanes:EtOAc) gave *N*-methoxy-*N*-methylbenzofuran-2-carboxamide (6.32 g, 100%). ^1^H NMR (CDCl_3_) *δ* 7.69 (ddd, 7.9, 1.2, 0.7 Hz, 1H), 7.61 (ddd, *J* = 8.4, 1.7, 0.9 Hz, 1H), 7.51 (d, *J* = 1.0 Hz, 1H), 7.48 (ddd, *J* = 7.9, 7.2, 1.3 Hz, 1H), 7.30 (ddd, *J* = 7.5, 7.3, 0.9 Hz, 1H), 3.84 (s, 3H), 3.43 (s, 3H). Found: [M+H] = 206.2.

Vinylmagnesium bromide (58 mL, 58 mmol, 1 M in THF) was added to a solution of the above amide (3.95 g, 19.2 mmol) in THF (200 mL, anhydrous) at 0 °C, the brown solution was warmed to 20 °C for 1 h then dimethylamine (58 mL, 116 mmol, 2 M in THF) and water (40 mL) were added. The solution was stirred at 20 °C for 1 h, and then partitioned between EtOAc and water. The solution was dried and evaporated to give 1-(benzofuran-2-yl)-3-(dimethylamino)propan-1-one (4.17 g, 100%). ^1^H NMR (CDCl_3_) *δ* 7.72 (ddd, *J* = 7.9, 1.0, 0.8 Hz, 1H), 7.59 (dd, *J* = 8.4, 0.8 Hz, 1H), 7.53 (d, *J* = 0.9 Hz, 1H), 7.48 (ddd, *J* = 7.8, 7.2, 1.2 Hz, 1H), 7.32 (ddd, *J* = 7.5, 7.2, 1.0 Hz, 1H), 3.14 (t, *J* = 7.5 Hz, 2H), 2.81 (t, *J* = 7.5 Hz, 2H), 2.31 (s, 6H). Found: [M+H] = 218.2.

##### 4.1.2.3 1-(Benzo[*b*]thiophen-7-yl)-3-(dimethylamino)propan-1-one (class B)

A suspension of 2-mercaptobenzoic acid (10.00 g, 6.49 mmol) in EtOH (50 mL) was treated with 2-bromo-1,1,-dimethoxyethane (10 mL, 8.5 mmol) and NaOH (5.70 g, 14.3 mmol) and the mixture was refluxed for 3 h. The solvent was evaporated and the residue was dissolved in DMF (100 mL), MeI (6.0 mL, 9.6 mmol) and K_2_CO_3_ (27.0 g, 19.5 mmol) were added and the mixture was stirred at 20 °C for 1 h, then partitioned between EtOAc and water, the organic layer was washed with water and brine, dried and evaporated. The residue was dissolved in chlorobenzene (50 mL), polyphosphoric acid (33 g) was added and the mixture was heated to 130 °C for 2 h. The gummy residue was poured onto ice and extracted with EtOAc, the organic fractions were washed with water, brine, dried and evaporated. Column chromatography with 10:1 hexanes:EtOAc gave methyl benzo[*b*]thiophene-7-carboxylate (6.46 g, 52%). ^1^H NMR (CDCl_3_) *δ* 8.12 (ddd, *J* = 6.9, 1.0, 0.4 Hz, 1H), 8.03 (dd, *J* = 7.9, 1.2 Hz, 1H), 7.58 (dd, *J* = 5.6, 0.3 Hz, 1H), 7.46 (t, *J* = 7.6 Hz, 1H), 7.40 (d, *J* = 5.6 Hz, 1H), 4.03 (s, 3H).

A solution of LiOH (2.10 g, 87.7 mmol) in water (25 mL) was added to a solution of the above ester (5.61 g, 29.2 mmol) in THF (50 mL) and MeOH (50 mL) and the solution was stirred at 20 °C for 18 h then evaporated. The residue was dissolved in water (150 mL) and acidified to pH 2 with conc. HCl. The precipitate was extracted into EtOAc, the organic fractions were dried and evaporated to give benzo[*b*]thiophene-7-carboxylic acid (4.69 g, 90%). ^1^H NMR (DMSO‑*d*_6_) *δ* 13.42 (s, 1H), 8.16 (d, *J* = 7.8 Hz, 1H), 8.04 (d, *J* = 7.4 Hz, 1H), 7.86 (d, *J* = 5.6 Hz, 1H), 7.50–7.56 (m, 2H). Found: [M−H] = 177.1.

Oxalyl chloride (2.67 mL, 31.6 mmol) was added to a suspension of the above acid (4.69 g, 26.3 mmol) in DCM (200 mL, anhydrous) and DMF (0.5 mL, 6.5 mmol) at 20 °C, stirred for 1 h then cooled to 0 °C. *N*,*O*-Dimethylhydroxylamine hydrochloride (3.08 g, 31.6 mmol) and pyridine (6.38 mL, 78.9 mmol) were added sequentially and the mixture was stirred at 20 °C for 18 h, then partitioned between EtOAc and sat. aq. NaHCO_3_. Column chromatography with DCM gave *N*-methoxy-*N*-methylbenzo[*b*]thiophene-7-carboxamide (5.48 g, 94%). ^1^H NMR (CDCl_3_) *δ* 7.92 (dd, *J* = 7.9, 1.0 Hz, 1H), 7.81 (dd, *J* = 7.5, 0.5 Hz, 1H), 7.53 (dd, *J* = 5.5, 0.3 Hz, 1H), 7.41 (t, *J* = 7.8 Hz, 1H), 7.37 (d, *J* = 5.5 Hz, 1H), 3.61 (s, 3H), 3.43 (s, 3H). Found: [M+H] = 222.1.

Vinylmagnesium bromide (49 mL, 1 M, 49 mmol) was added to a solution of the above amide (5.38 g, 24.3 mmol) in THF (250 mL, dist. Na) at 0 °C, the brown solution was warmed to 20 °C for 1 h and then dimethylamine in THF (49 mL, 2 M, 98 mmol) and water (25 mL) were added. The solution was stirred at 20 °C for 1 h, then partitioned between EtOAc and water. The solution was dried and evaporated to give 1-(benzo[*b*]thiophen-7-yl)-3-(dimethylamino)propan-1-one (5.45 g, 96%). ^1^H NMR (CDCl_3_) *δ* 8.07 (d, *J* = 7.8 Hz, 2H), 7.64 (d, *J* = 5.6 Hz, 1H), 7.50 (t, *J* = 7.6 Hz, 1H), 7.41 (d, *J* = 5.6 Hz, 1H), 3.34 (t, *J* = 7.7 Hz, 2H), 2.87 (t, *J* = 7.7 Hz, 2H), 2.33 (s, 6H). Found: [M+H] = 234.1.

##### 4.1.2.4 1-(Benzofuran-5-yl)-3-(dimethylamino)propan-1-one (class D-5)

A mixture of 5-bromobenzofuran (4.00 g, 20.3 mmol), DPPP (0.42 g, 1 mmol), triethylamine (6.3 mL, 45 mmol) and Pd(OAc)_2_ (0.23 g, 1 mmol) in DMSO (30 mL) and MeOH (30 mL) in a Berghof pressure reactor was evacuated then purged three times with carbon monoxide. The mixture was heated to 80 °C for 18 h under 60 psi of carbon monoxide pressure, cooled and partitioned between EtOAc and water. Column chromatography with 3:1 hexanes:DCM eluted traces of impurities while elution with DCM gave methyl benzofuran-5-carboxylate (2.77 g, 78%). ^1^H NMR (CDCl_3_) *δ* 8.35 (d, *J* = 1.4 Hz, 1H), 8.03 (dd, *J* = 8.7, 1.7 Hz, 1H), 7.69 (d, *J* = 2.2 Hz, 1H), 7.53 (dt, *J* = 8.7, 0.8 Hz, 1H), 6.84 (dd, *J* = 2.2, 1.0 Hz, 1H), 3.94 (s, 3H).

A solution of LiOH (1.13 g, 47.2 mmol) in water (20 mL) was added to a solution of the above ester (2.77 g, 15.7 mmol) in THF (40 mL) and MeOH (40 mL) and the solution was stirred at 20 °C for 18 h and then evaporated. The residue was dissolved in water (50 mL) and acidified with conc. HCl to pH 2. The precipitate was dissolved in EtOAc, the organic fraction was dried and evaporated to give benzofuran-5-carboxylic acid (2.49 g, 98%). ^1^H NMR (DMSO‑*d*_6_) *δ* 12.86 (s, 1H), 8.30 (d, *J* = 1.4 Hz, 1H), 8.10 (d, *J* = 2.2 Hz, 1H), 7.92 (dd, *J* = 8.6, 1.8 Hz, 1H), 7.68 (dt, *J* = 8.6, 0.7 Hz, 1H), 7.08 (dd, *J* = 2.2, 0.9 Hz, 1H).

Oxalyl chloride (1.55 mL, 18.3 mmol) was added to a suspension of the above acid (2.48 g, 15.3 mmol) in DCM (100 mL, anhydrous) and DMF (0.05 mL, 0.64 mmol) at 20 °C. The mixture was stirred at 20 °C for 1 h to give a colourless solution which was cooled to 0 °C. *N*,*O*-Dimethylhydroxylamine hydrochloride (1.64 g, 16.8 mmol) and pyridine (3.71 mL, 45.9 mmol) were added sequentially and the mixture was stirred at 20 °C for 18 h, then partitioned between EtOAc and sat. aq. NaHCO_3_. Column chromatography with 95:5 DCM:EtOAc gave *N*-methoxy-*N*-methylbenzofuran-5-carboxamide (2.28 g, 73%) as a pale brown oil. ^1^H NMR (CDCl_3_) *δ* 7.99 (d, *J* = 1.4 Hz, 1H), 7.65–7.69 (m, 2H), 7.52 (dt, *J* = 8.6, 0.6 Hz, 1H), 6.82 (dd, *J* = 2.2, 0.9 Hz, 1H), 3.56 (s, 3H), 3.39 (s, 3H). Found: [M+H] = 206.2.

Vinylmagnesium bromide (33.2 mL, 1 M in THF, 33.2 mmol) was added to a solution of the above anide (2.27 g, 11.1 mmol) in THF (100 mL, dist. Na) at 0 °C, the brown solution was stirred at 0 °C for 1 h then dimethylamine (33.2 mL, 2 M in THF, 66.4 mmol) and water (20 mL) were added. The solution was stirred at 20 °C for 1 h, and then partitioned between EtOAc and water. The solution was dried and evaporated to give 1-(benzofuran-5-yl)-3-(dimethylamino)propan-1-one (2.33 g, 97%). ^1^H NMR (CDCl_3_) *δ* 8.27 (d, *J* = 1.8 Hz, 1H), 7.98 (dd, *J* = 8.7, 1.8 Hz, 1H), 7.70 (d, *J* = 2.2 Hz, 1H), 7.55 (d, *J* = 8.7 Hz, 1H), 6.84 (dd, *J* = 2.2, 0.8 Hz, 1H), 3.22 (t, *J* = 7.1 Hz, 2H), 2.80 (t, *J* = 7.1 Hz, 2H), 2.31 (s, 6H). Found: [M+H] = 218.2.

##### 4.1.2.5 1-(Benzofuran-4-yl)-3-(dimethylamino)propan-1-one (class D-4)

A solution of benzofuran-4-ol (2.61 g, 19.5 mmol), DMAP (0.060 g, 0.49 mmol) and pyridine (2.37 mL, 29.3 mmol) in DCM (60 mL, anhydrous) at 0 °C was treated with TFAA (4.92 mL, 29.2 mmol) and then stirred at 0 °C for 2 h. The mixture was partitioned between DCM and water; the organic fractions were dried and evaporated. Column chromatography (hexanes) gave benzofuran-4-yl trifluoromethanesulfonate (3.93 g, 76%). ^1^H NMR (CDCl_3_) *δ* 7.70 (d, *J* = 2.3 Hz, 1H), 7.56 (d, *J* = 8.2 Hz, 1H), 7.35 (t, *J* = 8.2 Hz, 1H), 7.20 (d, *J* = 8.1 Hz, 1H), 6.90 (dd, *J* = 1.9, 1.0 Hz, 1H). Found: [M+H] = 267.0.

A mixture of the above sulfonate (3.88 g, 14.6 mmol), DPPP (0.180 g, 0.44 mmol), triethylamine (4.07 mL, 29.2 mmol) and Pd(OAc)_2_ (0.098 g, 0.44 mmol) in DMSO (50 mL) and MeOH (50 mL) in a Berghof pressure reactor was evacuated then purged three times with carbon monoxide. The mixture was heated to 80 °C for 18 h under 60 psi of carbon monoxide pressure, cooled and partitioned between EtOAc and water. Column chromatography with 3:1 hexanes:DCM eluted traces of impurities while elution with DCM gave methyl benzofuran-4-carboxylate (2.08 g, 81%). ^1^H NMR (CDCl_3_) *δ* 7.99 (dd, *J* = 7.7, 0.9 Hz, 1H), 7.74 (d, *J* = 2.2 Hz, 1H), 7.70 (dt, *J* = 8.2, 0.9 Hz, 1H), 7.33–7.39 (m, 2H), 3.99 (s, 3H).

A solution of LiOH (1.44 g, 34.3 mmol) in water (20 mL) was added to a solution of the above ester (2.02 g, 11.4 mmol) in THF (20 mL) and MeOH (20 mL) and the solution was stirred at 20 °C for 16 h and then evaporated. The residue was dissolved in water (50 mL) and acidified with conc. HCl and the precipitate was filtered and dried to give benzofuran-4-carboxylic acid (1.83 g, 99%). ^1^H NMR (DMSO‑*d*_6_) *δ* 13.10 (s, 1H), 8.14 (d, *J* = 2.1 Hz, 1H), 7.85–7.91 (m, 2H), 7.43 (t, *J* = 7.9 Hz, 1H), 7.33 (dd, *J* = 2.1, 1.0 Hz, 1H). Found: [M−H] = 161.1.

Oxalyl chloride (1.14 mL, 13.5 mmol) was added to a suspension of thew above acid (1.82 g, 11.2 mmol) in DCM (100 mL, anhydrous) and DMF (0.2 mL, 2.5 mmol) at 20 °C. The mixture was stirred at 20 °C for 1 h to give a colourless solution which was cooled to 0 °C. *N*,*O*-Dimethylhydroxylamine hydrochloride (1.31 g, 13.5 mmol) and pyridine (2.72 mL, 33.6 mmol) were added sequentially and the mixture was stirred at 20 °C for 18 h, then partitioned between DCM and water. Column chromatography with 95:5 DCM:EtOAc gave *N*-methoxy-*N*-methylbenzofuran-4-carboxamide (2.13 g, 93%). ^1^H NMR (CDCl_3_) *δ* 7.68 (d, *J* = 2.2 Hz, 1H), 7.59 (dt, *J* = 8.3, 0.8 Hz, 1H), 7.51 (dd, *J* = 7.5, 0.9 Hz, 1H), 7.32 (t, *J* = 7.9 Hz, 1H), 6.95 (dd, *J* = 2.2, 0.9 Hz, 1H), 3.56 (s, 3H). 3.40 (s, 3H). Found: [M+H] = 206.2.

Vinylmagnesium bromide (20.5 mL, 20.5 mmol) was added to a solution of the above amide (2.11 g, 10.3 mmol) in THF (100 mL, dist. Na) at 0 °C, the brown solution was warmed to 20 °C for 1 h, then dimethylamine in THF (20.5 mL, 2 M, 41 mmol) and water (20 mL) were added. The solution was stirred at 20 °C for 1 h, then partitioned between EtOAc and water. The solution was dried and evaporated to give 1-(benzofuran-4-yl)-3-(dimethylamino)propan-1-one (1.83 g, 82%). ^1^H NMR (CDCl_3_) *δ* 7.86 (dd, *J* = 7.6, 0.6 Hz, 1H), 7.76 (d, *J* = 2.1 Hz, 1H), 7.71 (dt, *J* = 8.2, 0.8 Hz, 1H), 7.55 (dd, *J* = 2.2, 1.0 Hz, 1H), 7.37 (t, *J* = 7.9 Hz, 1H), 3.27 (t, *J* = 7.1 Hz, 2H), 2.82 (t, *J* = 7.1 Hz, 2H), 2.32 (s, 6H). Found: [M+H] = 218.2.

##### 4.1.2.6 1-(Benzo[*b*]thiophen-7-yl)-3-(dimethylamino)propan-1-one (class D-7)

A suspension of 2-mercaptobenzoic acid (10.00 g, 6.49 mmol) in EtOH (50 mL) was treated with 2-bromo-1,1,-dimethoxyethane (10 mL, 8.5 mmol) and NaOH (5.70 g, 14.3 mmol) and the mixture was refluxed for 3 h. The solvent was evaporated and the residue was dissolved in DMF (100 mL), MeI (6.0 mL, 9.6 mmol) and K_2_CO_3_ (27.0 g, 19.5 mmol) were added and the mixture was stirred at 20 °C for 1 h, then partitioned between EtOAc and water, the organic layer was washed with water and brine, dried and evaporated. The residue was dissolved in chlorobenzene (50 mL), polyphosphoric acid (33 g) was added and the mixture was heated to 130 °C for 2 h. The gummy residue was poured onto ice and extracted with EtOAc, the organic fractions were washed with water, brine, dried and evaporated. Column chromatography with 10:1 hexanes:EtOAc gave methyl benzo[*b*]thiophene-7-carboxylate (6.46 g, 52%). ^1^H NMR (CDCl_3_) *δ* 8.12 (ddd, *J* = 6.9, 1.0, 0.4 Hz, 1H), 8.03 (dd, *J* = 7.9, 1.2 Hz, 1H), 7.58 (dd, *J* = 5.6, 0.3 Hz, 1H), 7.46 (t, *J* = 7.6 Hz, 1H), 7.40 (d, *J* = 5.6 Hz, 1H), 4.03 (s, 3H).

A solution of LiOH (2.10 g, 87.7 mmol) in water (25 mL) was added to a solution of the above ester (5.61 g, 29.2 mmol) in THF (50 mL) and MeOH (50 mL) and the solution was stirred at 20 °C for 18 h then evaporated. The residue was dissolved in water (150 mL) and acidified to pH 2 with conc. HCl. The precipitate was extracted into EtOAc, the organic fractions were dried and evaporated to give benzo[*b*]thiophene-7-carboxylic acid (4.69 g, 90%). ^1^H NMR (DMSO‑*d*_6_) *δ* 13.42 (s, 1H), 8.16 (d, *J* = 7.8 Hz, 1H), 8.04 (d, *J* = 7.4 Hz, 1H), 7.86 (d, *J* = 5.6 Hz, 1H), 7.50–7.56 (m, 2H). Found: [M−H] = 177.1.

Oxalyl chloride (2.67 mL, 31.6 mmol) was added to a suspension of the above acid (4.69 g, 26.3 mmol) in DCM (200 mL, anhydrous) and DMF (0.5 mL, 6.5 mmol) at 20 °C. The mixture was stirred at 20 °C for 1 h then cooled to 0 °C. *N*,*O*-Dimethylhydroxylamine hydrochloride (3.08 g, 31.6 mmol) and pyridine (6.38 mL, 78.9 mmol) were added sequentially and the mixture was stirred at 20 °C for 18 h, then partitioned between EtOAc and sat. aq. NaHCO_3_. Column chromatography with DCM gave *N*-methoxy-*N*-methylbenzo[*b*]thiophene-7-carboxamide (5.48 g, 94%). ^1^H NMR (CDCl_3_) *δ* 7.92 (dd, *J* = 7.9, 1.0 Hz, 1H), 7.81 (dd, *J* = 7.5, 0.5 Hz, 1H), 7.53 (dd, *J* = 5.5, 0.3 Hz, 1H), 7.41 (t, *J* = 7.8 Hz, 1H), 7.37 (d, *J* = 5.5 Hz, 1H), 3.61 (s, 3H), 3.43 (s, 3H). Found: [M+H] = 222.1.

Vinylmagnesium bromide (49 mL, 1 M, 49 mmol) was added to a solution of the above amide (5.38 g, 24.3 mmol) in THF (250 mL, dist. Na) at 0 °C, the brown solution was warmed to 20 °C over 1 h and then dimethylamine in THF (49 mL, 2 M, 98 mmol) and water (25 mL) were added. The solution was stirred at 20 °C for 1 h, then partitioned between EtOAc and water. The solution was dried and evaporated to give 1-(benzo[*b*]thiophen-7-yl)-3-(dimethylamino)propan-1-one (5.45 g, 96%). ^1^H NMR (CDCl_3_) *δ* 8.07 (d, *J* = 7.8 Hz, 2H), 7.64 (d, *J* = 5.6 Hz, 1H), 7.50 (t, *J* = 7.6 Hz, 1H), 7.41 (d, *J* = 5.6 Hz, 1H), 3.34 (t, *J* = 7.7 Hz, 2H), 2.87 (t, *J* = 7.7 Hz, 2H), 2.33 (s, 6H). Found: [M+H] = 234.1.

##### 4.1.2.7 1-(Benzofuran-5-yl)-3-(dimethylamino)propan-1-one (class D-5)

A mixture of 5-bromobenzofuran (4.00 g, 20.3 mmol), DPPP (0.42 g, 1 mmol), triethylamine (6.3 mL, 45 mmol) and Pd(OAc)_2_ (0.23 g, 1 mmol) in DMSO (30 mL) and MeOH (30 mL) in a Berghof pressure reactor was evacuated then purged three times with carbon monoxide. The mixture was heated to 80 °C for 18 h under 60 psi of carbon monoxide pressure, cooled and partitioned between EtOAc and water. Column chromatography with 3:1 hexanes:DCM eluted traces of impurities while elution with DCM gave methyl benzofuran-5-carboxylate (2.77 g, 78%). ^1^H NMR (CDCl_3_) *δ* 8.35 (d, *J* = 1.4 Hz, 1H), 8.03 (dd, *J* = 8.7, 1.7 Hz, 1H), 7.69 (d, *J* = 2.2 Hz, 1H), 7.53 (dt, *J* = 8.7, 0.8 Hz, 1H), 6.84 (dd, *J* = 2.2, 1.0 Hz, 1H), 3.94 (s, 3H).

A solution of LiOH (1.13 g, 47.2 mmol) in water (20 mL) was added to a solution of the above ester (2.77 g, 15.7 mmol) in THF (40 mL) and MeOH (40 mL) and the solution was stirred at 20 °C for 18 h and then evaporated. The residue was dissolved in water (50 mL) and acidified with conc. HCl to pH 2. The precipitate was dissolved in EtOAc, the organic fraction was dried and evaporated to give benzofuran-5-carboxylic acid (2.49 g, 98%). ^1^H NMR (DMSO‑*d*_6_) *δ* 12.86 (s, 1H), 8.30 (d, *J* = 1.4 Hz, 1H), 8.10 (d, *J* = 2.2 Hz, 1H), 7.92 (dd, *J* = 8.6, 1.8 Hz, 1H), 7.68 (dt, *J* = 8.6, 0.7 Hz, 1H), 7.08 (dd, *J* = 2.2, 0.9 Hz, 1H).

Oxalyl chloride (1.55 mL, 18.3 mmol) was added to a suspension of the above acid (2.48 g, 15.3 mmol) in DCM (100 mL, anhydrous) and DMF (0.05 mL, 0.64 mmol) at 20 °C. The mixture was stirred at 20 °C for 1 h to give a colourless solution which was cooled to 0 °C. *N*,*O*-Dimethylhydroxylamine hydrochloride (1.64 g, 16.8 mmol) and pyridine (3.71 mL, 45.9 mmol) were added sequentially and the mixture was stirred at 20 °C for 18 h, then partitioned between EtOAc and sat. aq. NaHCO_3_. Column chromatography with 95:5 DCM:EtOAc gave *N*-methoxy-*N*-methylbenzofuran-5-carboxamide (2.28 g, 73%) as a pale brown oil. ^1^H NMR (CDCl_3_) *δ* 7.99 (d, *J* = 1.4 Hz, 1H), 7.65–7.69 (m, 2H), 7.52 (dt, *J* = 8.6, 0.6 Hz, 1H), 6.82 (dd, *J* = 2.2, 0.9 Hz, 1H), 3.56 (s, 3H), 3.39 (s, 3H). Found: [M+H] = 206.2.

Vinylmagnesium bromide (33.2 mL, 1 M in THF, 33.2 mmol) was added to a solution of the above amide (2.27 g, 11.1 mmol) in THF (100 mL, dist. Na) at 0 °C, the brown solution was stirred at 0 °C for 1 h then dimethylamine (33.2 mL, 2 M in THF, 66.4 mmol) and water (20 mL) were added. The solution was stirred at 20 °C for 1 h, and then partitioned between EtOAc and water. The solution was dried and evaporated to give 1-(benzofuran-5-yl)-3-(dimethylamino)propan-1-one (2.33 g, 97%). ^1^H NMR (CDCl_3_) *δ* 8.27 (d, *J* = 1.8 Hz, 1H), 7.98 (dd, *J* = 8.7, 1.8 Hz, 1H), 7.70 (d, *J* = 2.2 Hz, 1H), 7.55 (d, *J* = 8.7 Hz, 1H), 6.84 (dd, *J* = 2.2, 0.8 Hz, 1H), 3.22 (t, *J* = 7.1 Hz, 2H), 2.80 (t, *J* = 7.1 Hz, 2H), 2.31 (s, 6H). Found: [M+H] = 218.2.

Similarly were prepared from known acids:

*3-(Dimethylamino)-1-(1-methyl-1H-indol-6-yl)propan-1-one (class E-6):*

^1^H NMR (CDCl_3_) *δ* 8.05 (t, *J* = 0.6 Hz, 1H), 7.74 (dd, *J* = 8.4, 1.5 Hz, 1H), 7.61 (dd, *J* = 8.4, 0.5 Hz, 1H), 7.23 (d, *J* = 3.0 Hz, 1H), 6.52 (dd, *J* = 3.0, 0.8 Hz, 1H), 3.87 (s, 3H), 3.29 (t, *J* = 7.2 Hz, 2H), 2.84 (t, *J* = 7.7 Hz, 2H), 2.35 (s, 6H).

*3-(Dimethylamino)-1-(1-methyl-1H-indol-4-yl)propan-1-one (****III****; class E-4):*

^1^H NMR (CDCl_3_) *δ* 7.78 (dd, *J* = 7.5, 0.6 Hz, 1H), 7.55 (d, *J* = 8.1 Hz, 1H), 7.30–7.25 (m, 2H), 7.22 (d, *J* = 3.1 Hz, 1H), 3.84 (s, 3H), 3.29 (t, *J* = 7.2 Hz, 2H), 2.85 (t, *J* = 7.8 Hz, 2H), 2.33 (s, 6H).

*3-(Dimethylamino)-1-(1-methyl-1H-indol-2-yl)propan-1-one (****III****; class E-2):*

^1^H NMR (CDCl_3_) *δ* 7.71 (d, *J* = 8.0 Hz, 1H), 7.38 (d, *J* = 3.5 Hz, 2H), 7.33 (s, 1H), 7.17–7.14 (m, 1H), 4.08 (s, 3H), 3.16 (t, *J* = 7.1 Hz, 2H), 2.78 (t, *J* = 7.5 Hz, 2H), 2.31 (s, 6H). Found: [M+H] = 231.5.

*3-(Dimethylamino)-1-(1-methylindolin-6-yl)propan-1-one (****III****; class F-6):*

^1^H NMR (CDCl_3_) *δ* 7.30 (dd, *J* = 7.5, 1.5 Hz, 1H), 7.11 (d, *J* = 7.6 Hz, 1H), 7.02 (d, *J* = 1.4 Hz, 1H), 3.36 (t, *J* = 8.3 Hz, 2H), 3.11 (t, *J* = 7.2 Hz, 2H), 2.99 (t, *J* = 8.1 Hz, 2H), 2.80 (s, 3H), 2.74 (t, *J* = 7.2 Hz, 2H), 2.29 (s, 6H).

*1-([1,3]Dioxolo[4,5-b]pyridin-7-yl)-3-(dimethylamino)propan-1-one (****III****; class J):*

^1^H NMR (CDCl_3_) *δ* 7.72 (d, *J* = 5.7 Hz, 1H), 7.20 (d, *J* = 5.7 Hz, 1H), 6.20 (s, 2H), 3.14 (t, *J* = 7.0 Hz, 2H), 2.74 (t, *J* = 7.2 Hz, 2H), 2.28 (s, 6H).

*1-(2,3-Dihydro-[1,4]dioxino[2,3-b]pyridin-8-yl)-3-(dimethylamino)propan-1-one (****III****; class L):*

^1^H NMR (CDCl_3_) *δ* 7.86 (d, *J* = 5.0 Hz, 1H), 7.12 (d, *J* = 5.0 Hz, 1H), 4.51–4.49 (m, 2H), 4.37–4.35 (m, 2H), 3.15 (t, *J* = 7.1 Hz, 2H), 2.70 (t, *J* = 7.3 Hz, 2H), 2.26 (s, 6H).

*3-(Dimethylamino)-1-(furo[3,2-c]pyridin-4-yl)propan-1-one (****III****; class M):*

^1^H NMR (CDCl_3_) *δ* 8.57 (d, *J* = 5.5 Hz, 1H), 7.78 (d, *J* = 2.2 Hz, 1H), 7.62 (dd, *J* = 5.5, 1.0 Hz, 1H), 7.59 (dd, *J* = 2.2, 1.0 Hz, 1H), 3.50 (t, *J* = 7.1 Hz, 2H), 2.83 (t, *J* = 7.1 Hz, 2H), 2.31 (s, 6H). Found [M+H] = 219.

*3-(Dimethylamino)-1-(furo[2,3-c]pyridin-7-yl)propan-1-one (****III****; Class N):*

^1^H NMR (CDCl_3_) *δ* 8.50 (d, *J* = 5.1 Hz, 1H), 7.90 (d, *J* = 2.2 Hz, 1H), 7.60 (d, *J* = 5.1 Hz, 1H), 6.87 (d, *J* = 2.2 Hz, 1H), 3.50 (t, *J* = 7.1 Hz, 2H), 2.85 (t, *J* = 7.1 Hz, 2H), 2.31 (s, 6H). Found [M+H] = 219.

#### 4.1.3 Scheme 3B: synthesis of Mannich base C/D units via methylketones

##### 4.1.3.1 1-(Benzofuran-7-yl)-3-(dimethylamino)propan-1-one (**V**: class D-7)

A solution of 7-bromobenzofuran (2.05 g, 10.4 mmol) in dry THF (20 mL) was prepared. Approximately 4 mL of this solution was added to a flask containing magnesium (0.75 g, 30.9 mmol) and the mixture was agitated until an exothermic reaction occurred. The remaining solution was added and the mixture was refluxed for 1 h, cooled and transferred by cannula to a dry flask. The solution was cooled to 0 °C and acetaldehyde (0.70 mL, 12.3 mmol) was added, the mixture was stirred at 0 °C for 1 h then partitioned between EtOAc and water, the organic fractions were dried and evaporated. Column chromatography with hexanes:DCM (1:1) eluted non polar impurities, elution with DCM gave 1-(benzofuran-7-yl)ethan-1-ol (1.21 g, 72%). ^1^H NMR (CDCl_3_) *δ* 7.64 (d, *J* = 2.2 Hz, 1H), 7.52 (dd, *J* = 7.7, 1.2 Hz, 1H), 7.34 (bd, *J* = 7.3 Hz, 1H), 7.24 (t, *J* = 7.6 Hz, 1H), 6.79 (d, *J* = 2.2 Hz, 1H), 5.38 (td, *J* = 6.5, 4.8 Hz, 1H), 2.16 (d, *J* = 4.8 Hz, 1H), 1.67 (d, *J* = 6.5 Hz, 3H). Found: [M−H_2_O] = 145.

A mixture of the above alcohol (1.16 g, 7.15 mmol) and MnO_2_ (3.10 g, 35.6 mmol) in benzene (40 mL) was refluxed for 1 h, filtered through Celite and the solvent was evaporated. Column chromatography with hexanes:DCM (3:1–1:1) gave 1-(benzofuran-7-yl)ethan-1-one (0.98 g, 86%). ^1^H NMR (CDCl_3_) *δ* 7.92 (dd, *J* = 7.6, 1.2 Hz, 1H), 7.81 (dd, *J* = 7.7, 1.2 Hz, 1H), 7.75 (d, *J* = 2.2 Hz, 1H), 7.33 (t, *J* = 7.7 Hz, 1H), 6.87 (d, *J* = 2.2 Hz, 1H), 2.86 (s, 3H). Found: [M+H] = 161.1.

A mixture of the above methylketone (4.95 g, 30.9 mmol), dimethylamine HCl (3.78 g, 46.4 mmol) and paraformaldehyde (1.39 g, 46.3 mmol) in EtOH (50 mL) and HCl (0.5 mL, 12 M, 6 mmol) was refluxed in a sealed tube for 18 h. The solvent was evaporated and the solid residue was triturated with Et_2_O and filtered. The solid was washed with Et2O, dissolved in water and basified with 2 M NaOH, then extracted with EtOAc (3 × 100 mL). The organic fractions were dried and evaporated to give 1-(benzofuran-7-yl)-3-(dimethylamino)propan-1-one (4.63 g, 69%). ^1^H NMR (CDCl_3_) *δ* 7.93 (dd, *J* = 7.6, 1.1 Hz, 1H), 7.81 (dd, *J* = 7.7, 1.3 Hz, 1H), 7.74 (d, *J* = 2.2 Hz, 1H), 7.33 (t, *J* = 7.7 Hz, 1H), 6.87 (d, *J* = 2.2 Hz, 1H), 3.46 (t, *J* = 7.5 Hz, 2H), 2.82 (t, *J* = 7.5 Hz, 2H), 2.32 (s, 6H). Found: [M+H] = 218.2.

Similarly were prepared:

*3-(Dimethylamino)-1-(1-methylindolin-4-yl)propan-1-one (****V****; class E-4):*

^1^H NMR (CDCl_3_) *δ* 7.17 (m, 2H), 6.60 (t, *J* = 3.9 Hz, 1H), 3.38–3.28 (m, 4H), 3.09 (t, *J* = 7.1 Hz, 2H), 2.78 (s, 3H), 2.73 (t, *J* = 7.1 Hz, 2H), 2.28 (s, 6H).

*1-(Benzo[d][1,3]dioxol-4-yl)-3-(dimethylamino)propan-1-one (****V****; class H):*

^1^H NMR (CDCl_3_) *δ* 7.39 (dd, *J* = 8.2, 1.2 Hz, 1H), 6.98 (dd, *J* = 7.6, 1.2 Hz, 1H), 6.88 (t, *J* = 7.9 Hz, 1H), 6.08 (s, 2H), 3.15 (t, *J* = 7.4 Hz, 2H), 2.74 (t, *J* = 7.4 Hz, 2H), 2.28 (s, 6H). Found: [M+H] = 222.7.

*3-(Dimethylamino)-1-(furo[3,2-b]pyridin-7-yl)propan-1-one (****V****; class K):*

^1^H NMR (CDCl_3_) *δ* 8.70 (d, *J* = 5.0 Hz, 1H), 7.99 (d, *J* = 2.3 Hz, 1H), 7.67 (d, *J* = 5.0 Hz, 1H), 7.11 (d, *J* = 2.3 Hz, 1H), 3.46 (t, *J* = 6.9 Hz, 2H), 2.81 (t, *J* = 7.2 Hz, 2H), 2.31 (s, 6H). Found: [M+H] = 219.4.

#### 4.1.4 Scheme 1: preparation of the 6-bromo compounds of Table 1

##### 4.1.4.1 Example of 1-(6-bromo-2-methoxyquinolin-3-yl)-2-(2,3-dihydro-1H-inden-4-yl)-1-(2,3-dimethoxyphenyl)-4-(dimethylamino)butan-2-ol (**9**)

A solution of dry diisopropylamine (0.92 mL, 6.49 mmol) in dry THF (10 mL) was cooled to −40 °C under an atmosphere of dry nitrogen. *N*-Butyllithium (3.25 mL of a 2.0 N solution in cyclohexane, 6.49 mmol) was added dropwise, then stirring was continued for a further 15 min. The solution was cooled to −70 to −78 °C and a solution of 6-bromo-3-(2,3-dimethoxybenzyl)-2-methoxyquinoline^1^ (2.10 g, 5.41 mmol) in dry THF (6 mL) was added dropwise. The resulting purple solution was stirred at this temperature for 60 min. A solution of **19** (1.07 g, 4.92 mmol) in THF (6 mL) was added dropwise and the mixture was stirred at this temperature for 5 h. Glacial acetic acid (0.70 mL) was added in one portion and the mixture was allowed to warm to room temperature. Water was added and the mixture was extracted with ethyl acetate. The extract was washed with water and dried over sodium sulfate. Removal of the solvent under reduced pressure left an oil, which was chromatographed on silica. Column chromatography with hexanes:EtOAc (1:1) gave fore fractions, followed by isomer A of **9** (0.452 g, 14%). Elution with EtOAc gave isomer B of **9** (1.15 g, 35%).

Isomer A, pale yellow solid. ^1^H NMR (CDCl_3_, 400 MHz) *δ* 8.19 (s, 1H), 7.86–7.81 (m, 2H), 7.72–7.66 (m, 2H), 7.59 (dd, *J* = 8.8, 2.2 Hz, 1H), 7.08–6.98 (m, 3H), 6.82 (t, *J* = 8.1 Hz, 1H), 6.57 (dd, *J* = 8.2, 1.3 Hz, 1H), 5.91 (s, 1H), 4.24 (s, 3H), 3.65 (s, 3H), 3.34 (s, 3H), 3.30–3.21 (m, 1H), 3.10–2.98 (m, 1H), 2.77 (t, *J* = 7.3 Hz, 2H), 2.20–2.05 (m, 2H), 2.03–1.96 (m, 2H), 1.93 (s, 6H), 1.75–1.59 (m, 2H). Found: [M+H] =  605.7.

Isomer B, white solid. ^1^H NMR (CDCl_3_, 400 MHz) *δ* 8.79 (s, 1H), 7.95 (br s, 1H), 7.81 (s, 1H), 7.62 (d, *J* = 7.1 Hz, 1H), 7.52–7.45 (m, 2H), 7.28–7.23 (m, 1H), 6.99–6.92 (m, 3H), 6.79 (dd, *J* = 8.1, 1.4 Hz, 1H), 5.82 (s, 1H), 4.03 (s, 3H), 3.90 (s, 3H), 3.81 (s, 3H), 3.32–3.24 (m, 1H), 3.08–3.00 (m, 1H), 2.82–2.71 (m, 2H), 2.18–2.07 (m, 2H), 2.05–1.98 (m, 2H), 1.95 (s, 6H), 1.93–1.79 (m, 2H). Found: [M+H] =  605.7.

Each coupled product was resolved into its four optical isomers using preparative chiral HPLC at BioDuro LLC (Beijing).

#### 4.1.5 Scheme 1: preparation of the 6-cyano compounds of Table 1

##### 4.1.5.1 Example of 3-(2-(2,3-dihydro-1H-inden-4-yl)-1-(2,3-dimethoxyphenyl)-4-(dimethylamino)-2-hydroxybutyl)-2-methoxyquinoline-6-carbonitrile (**10**)

A solution of **9** (2.41 g, 3.98 mmol) in DMF (10 mL, anhydrous) was purged with nitrogen and heated to 55 °C for 10 min. Tri(*o*-tolyl)phosphine (0.242 g, 0.796 mmol), zinc dust (0.026 g, 0.398 mmol) and tris(dibenzylideneacetone)dipalladium(0) (0.364 g, 0.398 mmol) were then added, and the reaction was again purged with nitrogen and heated for another 10 min at 55 °C. Zinc cyanide (0.327 g, 2.79 mmol) was then added and the reaction mixture was heated to 65 °C for 4 h. The reaction was diluted with water and extracted with EtOAc three times. The organic layer was washed with brine three times, dried and evaporated. Column chromatography with 1:1 hexane/EtOAc followed by 4:1 hexane/EtOAc afforded isomer A of **10** (0.26 g, 12%) followed by isomer B of **10** (0.92 g, 42%) as white solids.

Isomer A, pale yellow solid. ^1^H NMR (CDCl_3_, 400 MHz) *δ* 8.30 (s, 1H), 8.05 (d, *J* = 1.8 Hz, 1H), 7.88–7.81 (m, 2H), 7.71–7.66 (m, 2H), 7.08–6.98 (m, 3H), 6.83 (t, *J* = 8.1 Hz, 1H), 6.58 (dd, *J* = 8.2, 1.3 Hz, 1H), 5.91 (s, 1H), 4.28 (s, 3H), 3.66 (s, 3H), 3.38 (s, 3H), 3.29–3.22 (m, 1H), 3.10–2.99 (m, 1H), 2.78 (t, *J* = 7.1 Hz, 2H), 2.20–2.06 (m, 2H), 2.03–1.96 (m, 2H), 1.92 (s, 6H), 1.75–1.60 (m, 2H). Found: [M+H] =  552.5.

Isomer B, white solid. ^1^H NMR (CDCl_3_, 400 MHz) *δ* 8.92 (s, 1H), 8.03 (d, *J* = 1.2 Hz, 1H), 7.66–7.58 (m, 3H), 7.25–7.22 (m, 1H), 7.00–6.93 (m, 4H), 6.81 (dd, *J* = 8.2, 1.4 Hz, 1H), 5.82 (s, 1H), 4.04 (s, 3H), 3.91 (s, 3H), 3.84 (s, 3H), 3.30–3.21 (m, 1H), 3.09–3.01 (m, 1H), 2.82–2.71 (m, 2H), 2.19–2.05 (m, 2H), 2.05–1.98 (m, 2H), 1.96 (s, 6H), 1.89–1.79 (m, 2H). Found: [M+H] = 552.5.

In each case, the coupled 6-cyano product was then resolved into its four optical isomers using preparative chiral HPLC at BioDuro LLC (Beijing).

## A Supplementary data

Supplementary data 1

Supplementary data 2 Additional biological data on the compounds of Table 2.

### MOL files

The following ZIP file contains the MOL files of the most important compounds referred to in this article.

MOL files ZIP file containing the MOL files of the most important compounds in this article.
