# Supplementary material for: Structure-activity relationships for analogs of the tuberculosis drug bedaquiline with the naphthalene unit replaced by bicyclic heterocycles
Source: Bioorg Med Chem. 2018 May 1;26(8):1797–809. doi: 10.1016/j.bmc.2018.02.026 (PMC5933462; doi:10.1016/j.bmc.2018.02.026)
Supplement: Supplementary data 2 — Additional biological data on the compounds of Table 2. [file mmc2.docx]

**Supplementary Material**

for

**Structure-activity relationships for analogues of the tuberculosis drug bedaquiline with the naphthalene unit replaced by bicyclic heterocycles**

Hamish S. Sutherland, Amy S.T. Tong, Peter J. Choi, Daniel Conole, Adrian Blaser, Scott G. Franzblau, Christopher B. Cooper, Anna M. Upton, Manisha U. Lotlikar, William A. Denny, Brian D. Palmer

Table S1. Additional ADME data for the compounds of Table 2

| No | Solubility^a^ | PPB^b^ | F^c^ | HLM^d^ | HLM +3A4 inhibitor^e^ | clogP^f^ |
| --- | --- | --- | --- | --- | --- | --- |
|  | µM | % | % | T½ (min) | T½ (min) |  |
| **1** | < 0.06 | 100 | 56 | 182 | 1733 | 7.25 |
| **5** | <0.06 | 100 | 33 | 36 | 47 | 7.11 |
| **7** | <0.06 | 100 | 54 | 87 | 990 | 7.09 |
| **10** | ND | ND | 25 | ND | ND | 4.99 |
| **11** | <0.06 | 100 | 51 | 62 | 347 | 7.09 |
| **14** | <0.06 | 100 | 50 | 46 | 108 | 5.28 |
| **16** | 0.3 | 99.997 | 42 | 54 | 630 | 6.64 |
| **18** | 0.13 | ND | 13 | 36 | 120 | 5.56 |
| **21** | ND | ND | ND | ND | ND | 6.48 |
| **22** | 0.15 | 99.86 | 37 | 53 | 267 | 5.61 |
| **24** | <0.06 | 99.99 | 49 | 224 | 315 | 7.41 |
| **25** | <0.06 | 100 | 21 | 10 | 154 | 6.06 |
| **26** | <0.02 | 100 | 73 | 161 | 248 | 6.87 |
| **27** | ND | ND | ND | ND | ND | 5.53 |
| **28** | 0.016 | 100 | 41 | 131 | 267 | 7.19 |
| **29** | <0.06 | 100 | 25 | 173 | NA | 5.84 |
| **30** | <0.02 | 100 | 50 | 204 | 154 | 7.42 |
| **34** | <0.06 | 100 | 42 | 22 | 42 | 6.64 |
| **36** | 0.22 | 100 | 52 | 42 | 96 | 5.61 |
| **37** | ND | ND | ND | ND | ND | 5.61 |
| **42** | <0.2 | 100 | 33 | 33 | 90 | 5.42 |
| **43** | <0.02 | 100 | 50 | 25 | 36 | 5.61 |
| **48** | ND | ND | ND | ND | ND | 6.5 |
| **50** | <0.2 | 98.6 | 35 | 51 | 347 | 6.5 |
| **54** | 11 | 99.43 | 21 | 29 | 578 | 3.75 |
| **55** | <0.06 | 100 | 28 | 88 | 770 | 6.54 |
| **56** | <0.2 | 99.98 | 38 | 61 | 578 | 5.18 |
| **57** | <0.2 | 100 | ND | 239 | NA | 6.71 |
| **60** | 64 | 99.96 | 61 | 71 | 133 | 4.97 |

Footnotes for Table S1: ^a^Kinetic aqueous solubility at pH 7.4 at 20^o^C; ethanolamine (5 mM), KH_2_PO_4_ (45mM), KOAc (45 mM), KCl (75 mM); ^b^To human plasma at 37 ^o^C; ^c^Oral bioavailability in mice; ^d,e^Clearance (µL/min/mg) in human liver microsomes at 60 min (1 µM concentration), without^d^ and with^e^ With 2 µM of CYP3A4 inhibitor ketoconazole; ^f^clogP calculated by ChemDraw Ultra v12.0.2. (CambridgeSoft).
